# Data on development of the Wistar rat skeleton at the end of gestation

**DOI:** 10.1016/j.dib.2019.104225

**Published:** 2019-07-06

**Authors:** Bernd Baier, Bruno Viertel

**Affiliations:** a Nonclinical Drug Safety Germany, Boehringer Ingelheim Pharma GmbH & Co. KG, Biberach an der Riss, Germany; b Trier University, Department of Biogeography, Universitätsring 15, D-54286, Trier, Germany

## Abstract

Data presented gives detailed information on bone development and ossification grade of Wistar rat fetuses close to parturition (Gestation days 21–23). Focus lies on the representation of the ossification status of an individual and options to summarize the data without loss of information. Moreover the body weight development and food consumption data of the dams and non-pregnant females is included. For further interpretation please refer to the related article “The ossification status of Wistar rat fetuses at the end of gestation” (1).

**Table:** Specifications table

Subject	Biology
Specific subject area	Reproductive Toxicology
Type of data	Figure Supplementary tables
How data were acquired	Examination of pregnant rats and offspring
Data format	Filtered Raw
Parameters for data collection	Animals were not test item treated and the controlled variable was the different gestation day of termination of pregnancy.
Description of data collection	Data was collected as part of an evaluation study, supporting regulatory reproductive and embryofetal toxicity studies for the qualification of pharmaceuticals. Female rats (144 animals) were mated and body weight development, food consumption and clinical signs were monitored. The pregnant rats were hysterectomized on different days of pregnancy (Gestation days 21–23). Status of all implants was recored and living fetuses further examined for external morphological abberations and the status of skeletal development.
Data source location	Not relevant
Data accessibility	With the article
Related research article	Associated research article: Bernd Baier, Bruno Viertel The ossification status of Wistar rat fetuses at the end of gestation. Reproductive Toxicology https://doi.org/10.1016/j.reprotox.2019.03.005

**Table:** 

**Value of the data** • Quantitative insight in the ossification process of Wistar rats at the end of gestation • Individual fetus data allows recognition of developmental patterns in bone development • Different approaches for graphical data presentation from reproductive toxicology studies are provided

## 1 Data

**Fig. 1:**
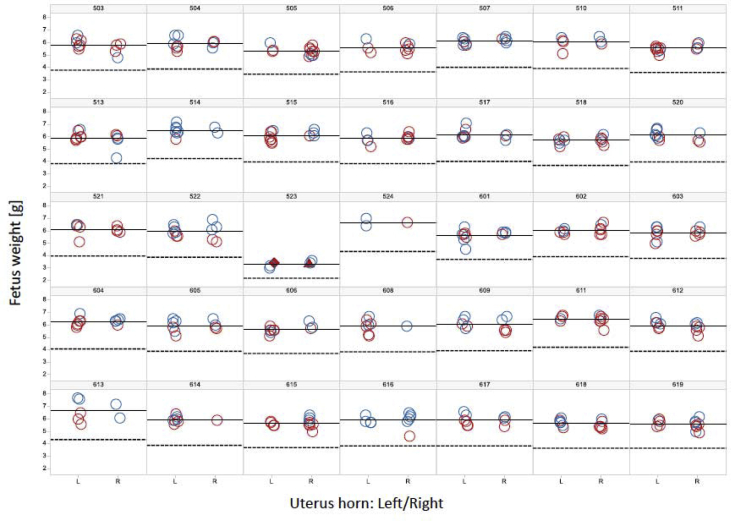
Body weight, sex and uterine position for fetuses from 30 litters on gestation day 23. Bold line gives mean litter weight, dashed line the 65% mean cutoff for runts. Color gives fetal sex, red female, blue males. Empty circles mark normal fetuses. Filled triangle marks fetuses with unossified 3rd and 6th cervical vertebral centrum. Filled diamond marks fetuses with unossified 6th *but ossified* 3rd cervical vertebral centrum. This deviation from the normal ossification pattern occurred in a grossly malformed fetuses (cleft palate) from one litter.

**Fig. 2:**
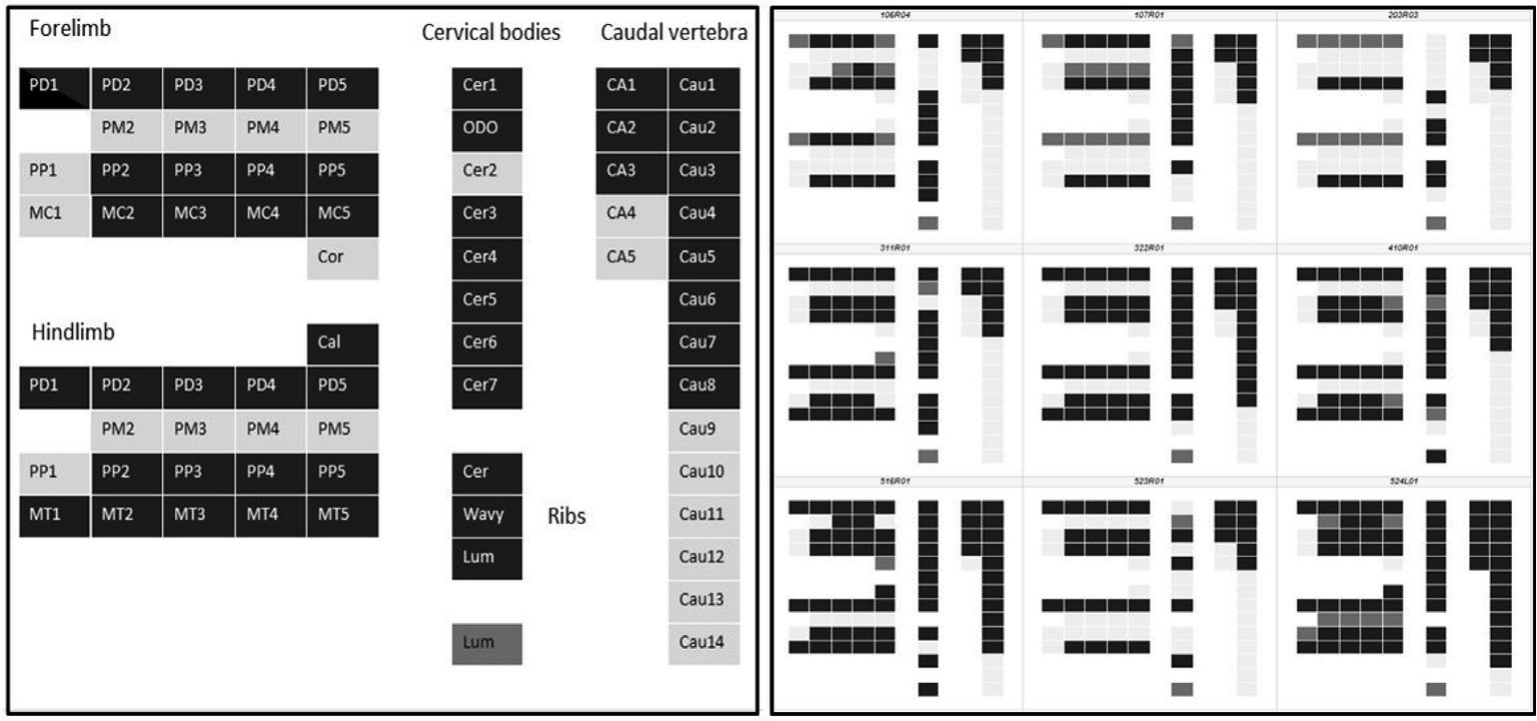
Graphical view of different bone elements. On the left a typical fetus on gestation day 23 is depicted to describe the bone elements under research. Shading grade codes ossification progress from lighter to darker as ossification increases. Abbreviations: PD#: phalanx distal, PM#: phalanx middle, PP#: phalanx proximal, MC#: metacarpal, Cor: processus coracoideus, Cal: calcaneus, MT#: metatarsal, Cer#: cervical body, ODO: odontoid, Cer: cervical rib, Wavy: wavy rib, Lum: processus transversus of 4th lumbar arch, CA#: caudal arch, Cau#: caudal body.

Data presented gives detailed insights on status of bone development of Wistar rat fetuses from over 140 litters close to parturition (Gestation days 21–23). A special focus lies on the presentation of co-occurring findings in single fetuses or litters. Co-occurring findings are not easily accessible in the usual table presentation of reproductive toxicology studies. Therefore, we used a graphical representation of each litter based on laterality in the uterus, fetal sex and body weight, allowing for filtering different fetal findings (Fig. 1). Additionally a graphical representation of the bone structures most commonly affected by deviations from the expected ossification status was prepared (Fig. 2). This allowed for interactive filtering for all other findings and supported recognition of patterns, otherwise not easily discernable.

**Fig. 3:**
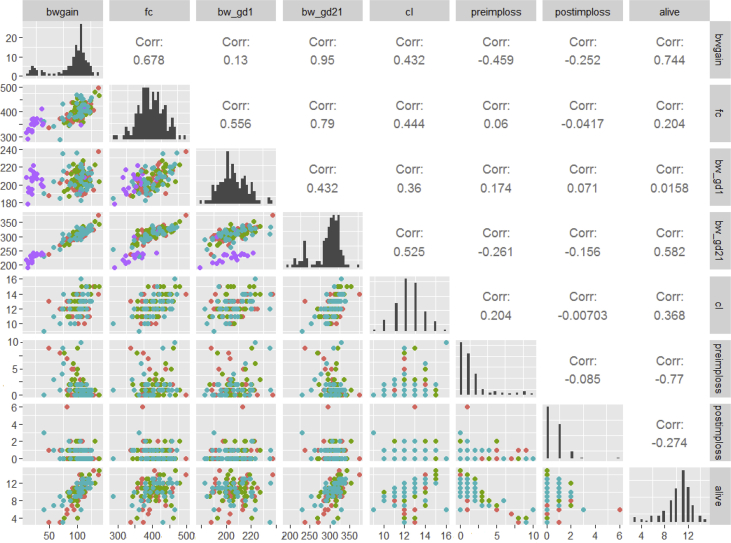
Data on females during pregnancy: body weight gain (bwgain), food consumption (fc), body weight on gestation day 1 (bw_gd1), body weight on gestation day 21 (bw_gd21), number of corpora lutea (cl), number of eggs not implanted (preimploss), number of implants that died in utero (postimploss) and living fetuses. Different colors code for the three different hysterectomy dates. Purple indicates non-pregnant females. On the right upper part coefficients of correlation are printed.

The influence of maternal parameters – especially weight development and food consumption, are relevant to determine if maternal effects have affected the pregnancy outcome. Therefore, we correlated these maternal in-life parameters with litter parameters observed (Fig. 3). Weight development and food consumption of non-pregnant females deviated from the pregnant animals, but otherwise no significant correlations between litter parameters and maternal parameters were present.

On the right side the top, middle and bottom row shows three fetuses from GD 21, 22 and 23, respectively. Note that the GD 23 fetus in the middle of the bottom row is the fetus with the abnormal ossification pattern in the cervical region (marked with the triangle in Fig. 1).

## 2 Experimental design, materials, and methods

Data was collected in the laboratory for reproductive toxicology at Boehringer Ingelheim Pharma GmbH & Co. KG. Methods, conduct and reporting complied with the standards of Good Laboratory Practice (GLP) and are described in detail in Baier and Viertel, 2019 [1]. In short, 144 Crl:WI(Han) female rats were mated at an age of about 12 weeks and treated daily with a 0.5% hydroxyethyl cellulose suspension in demineralized water by gavage at a dose volume of 10 mL/kg body weight from GD 7–15. Food and water were provided ad libitum to the single housed animals. Pregnancy was terminated on Gestation Days 21, 22, 23 to collect the fetuses and examine the status of skeletal development after staining with alizarin red S. Data was recorded on paper in a form for over 100 bone structures and additional anomalies were recored in free text. The data showing deviations from normal was manually digitized. Data presentations were prepared in the software package R (3.5.1) and Spotfire.

The raw data and additional graphical presentations are provided as electronic supplement to allow further analysis and deeper insights into the sequence of ossification during late pregnancy in Wistar rats.
