# Supplementary material for: Data on development of the Wistar rat skeleton at the end of gestation
Source: Data Brief. 2019 Jul 6;25:104225. doi: 10.1016/j.dib.2019.104225 (PMC6646921; doi:10.1016/j.dib.2019.104225)

GD 21 Litters 102, 103, 104, 105

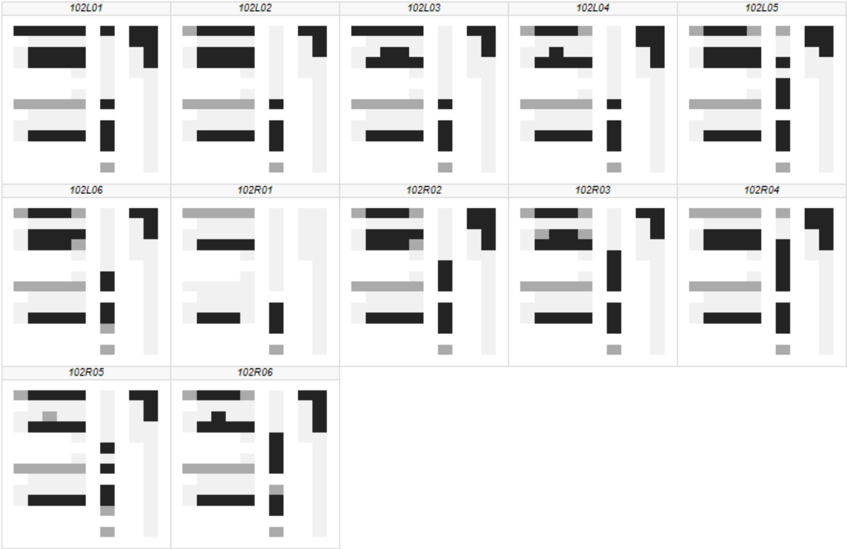

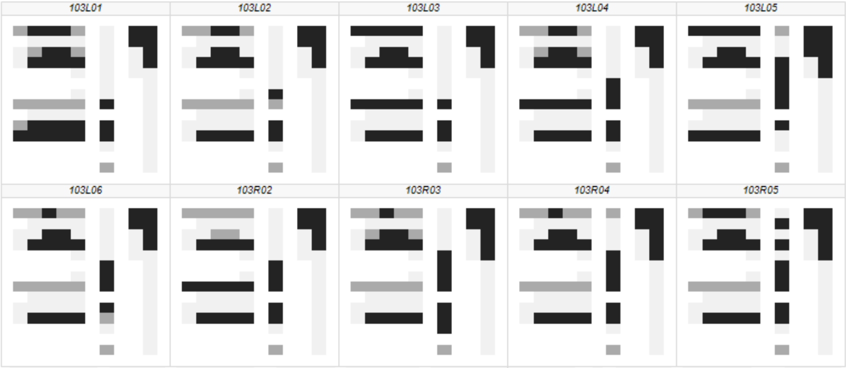

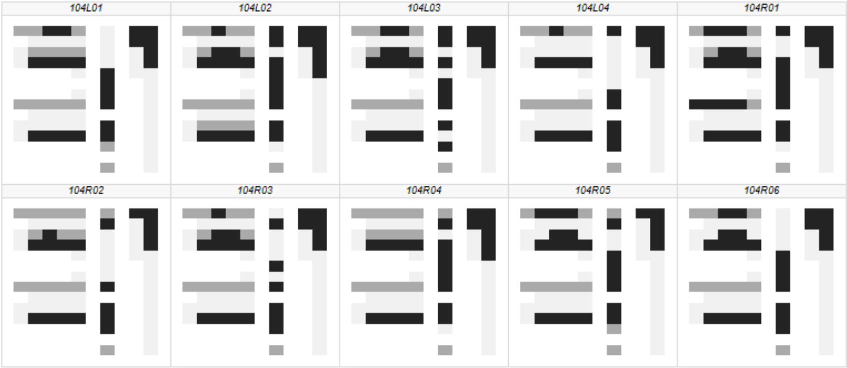

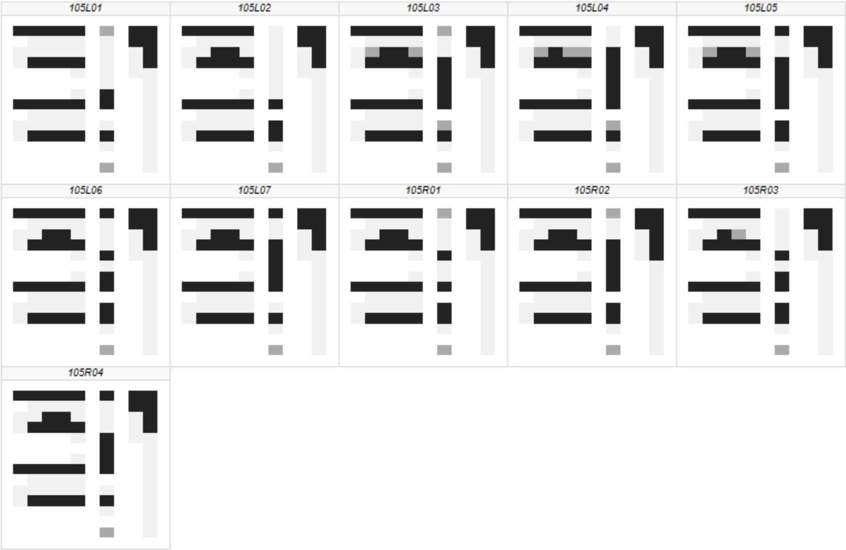

GD 21 Litters 106, 107, 108, 109

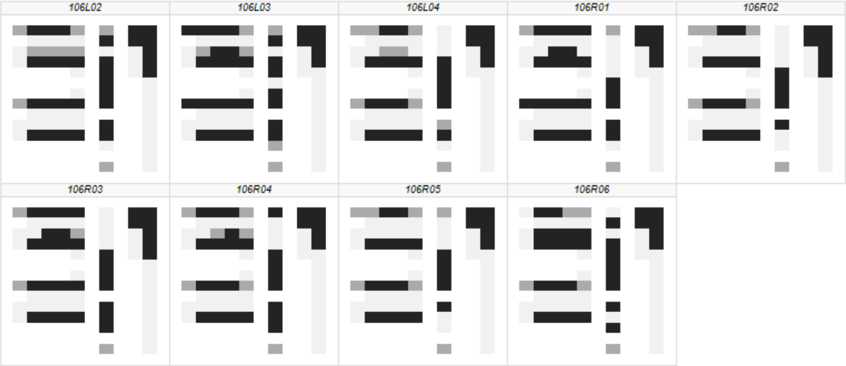

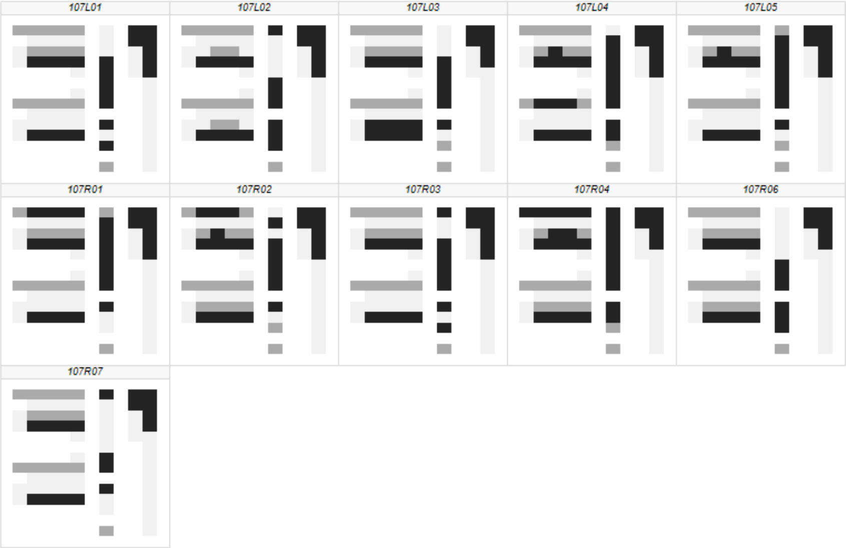

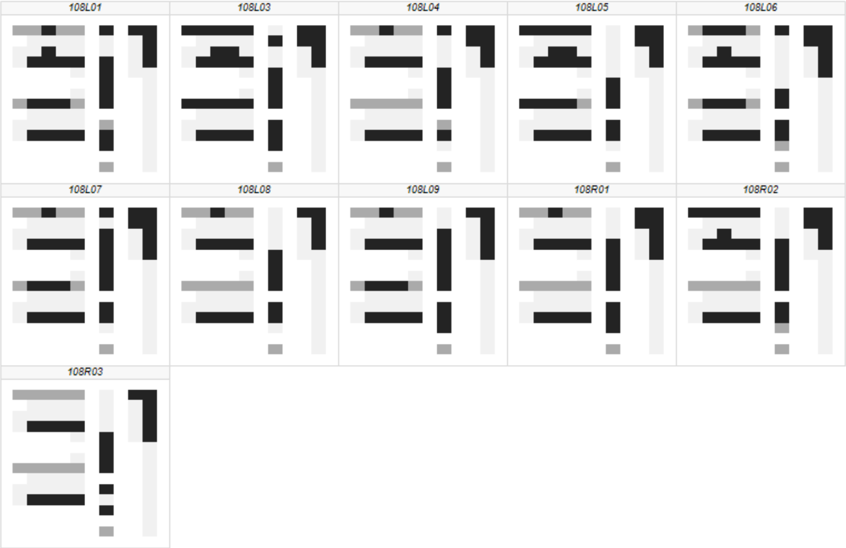

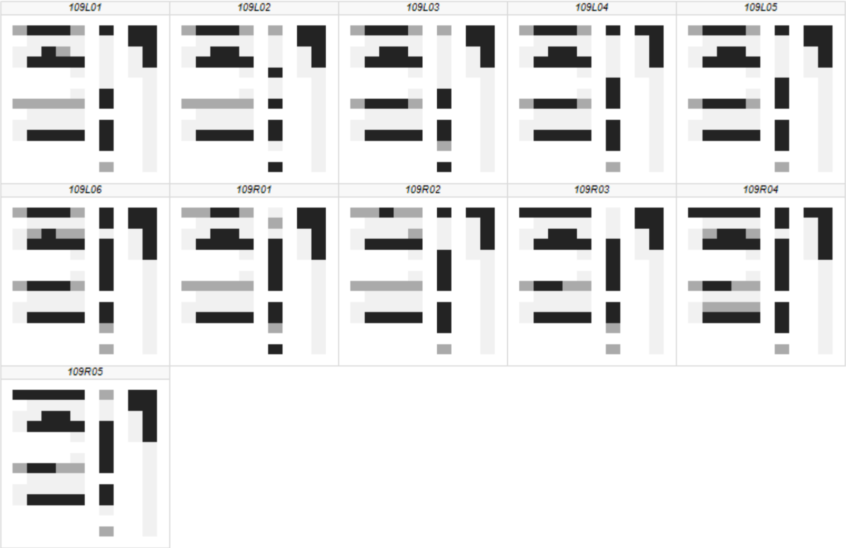

GD 21: Litters 110, 111, 112, 113

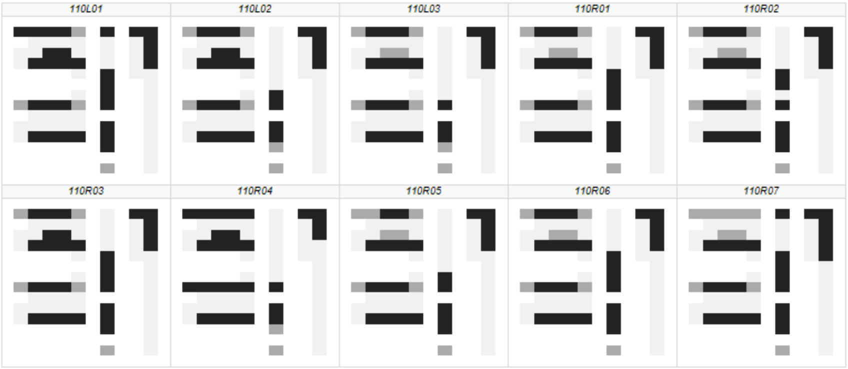

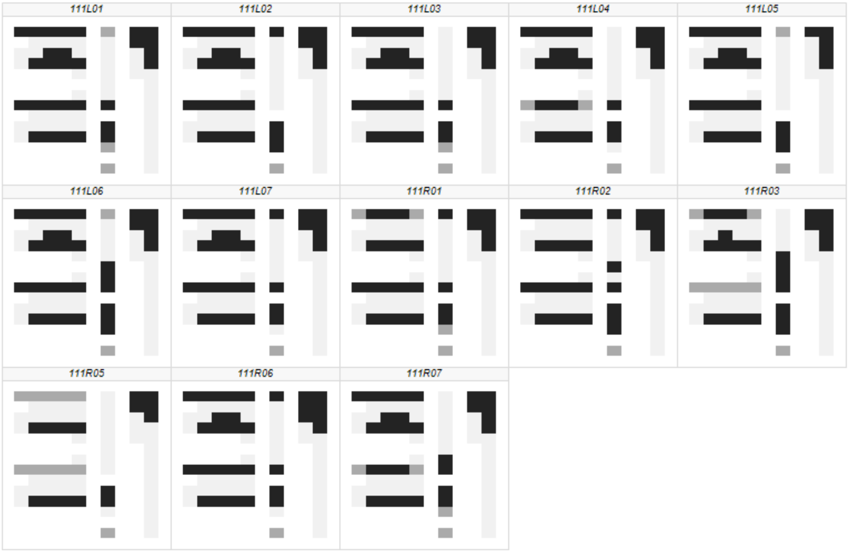

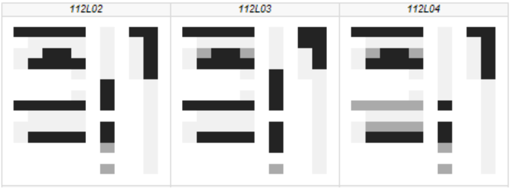

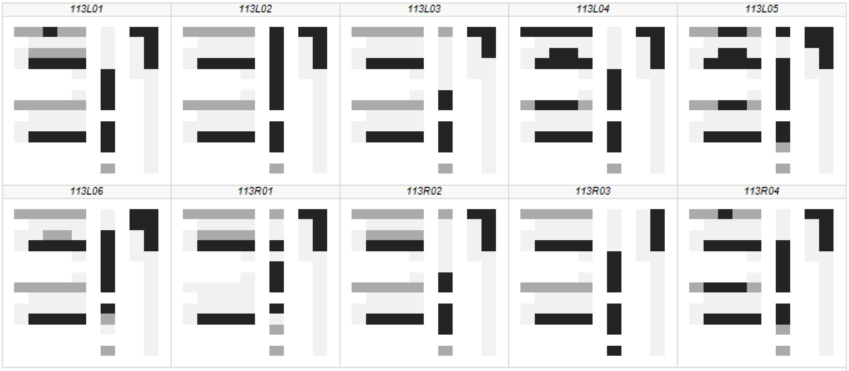

GD 21: Litters 115, 116, 117, 118

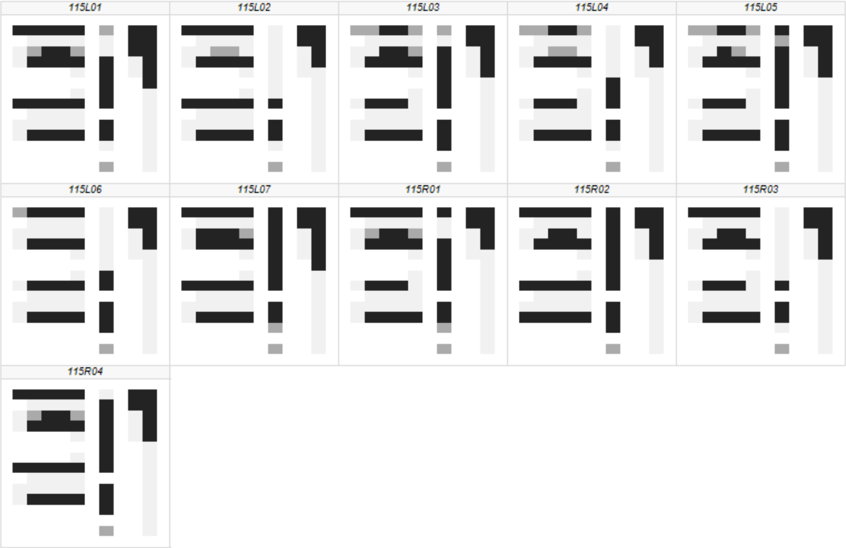

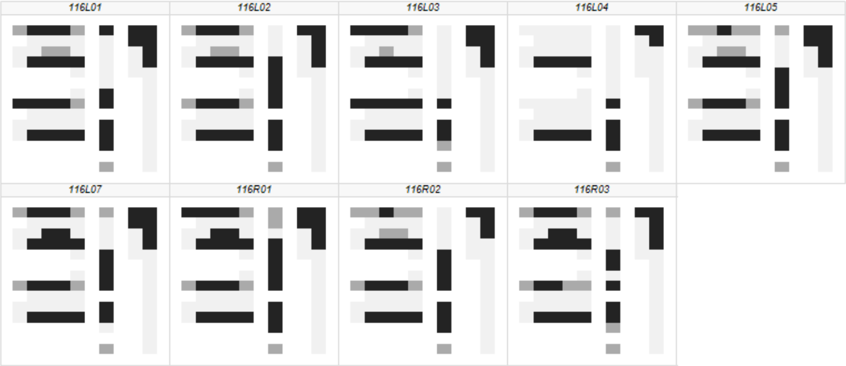

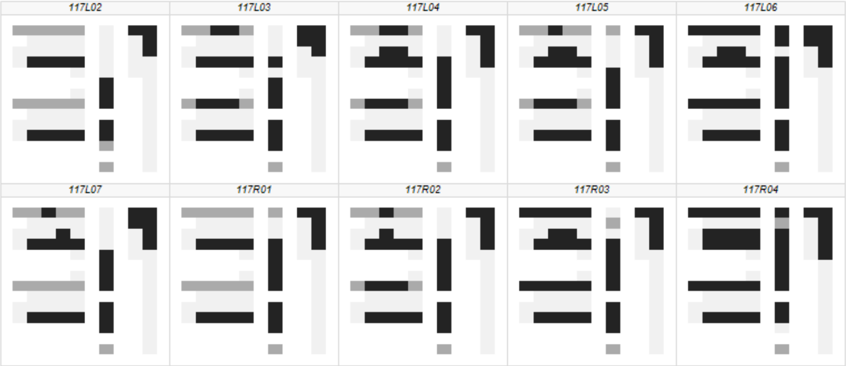

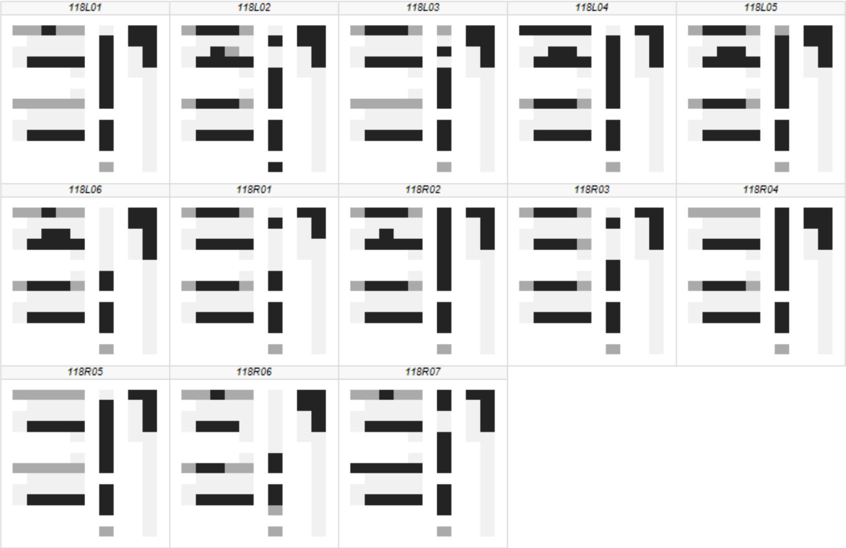

GD 21: Litters 120, 121, 123, 124

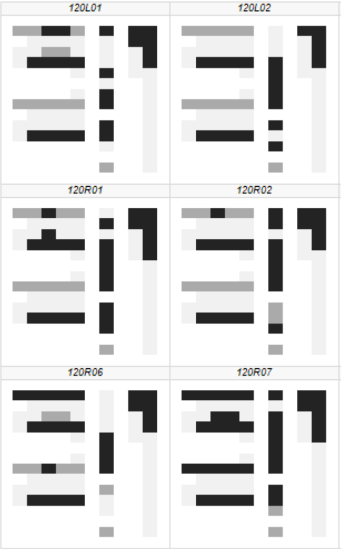

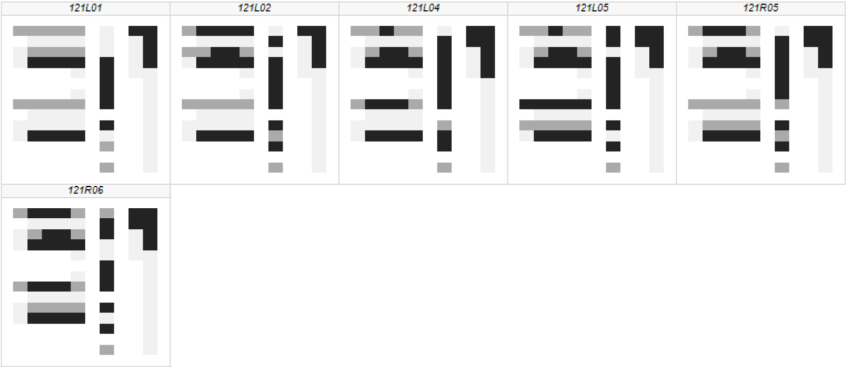

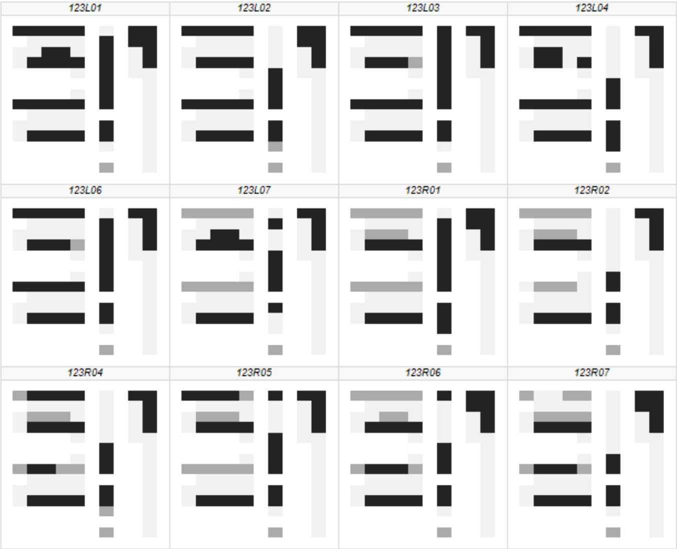

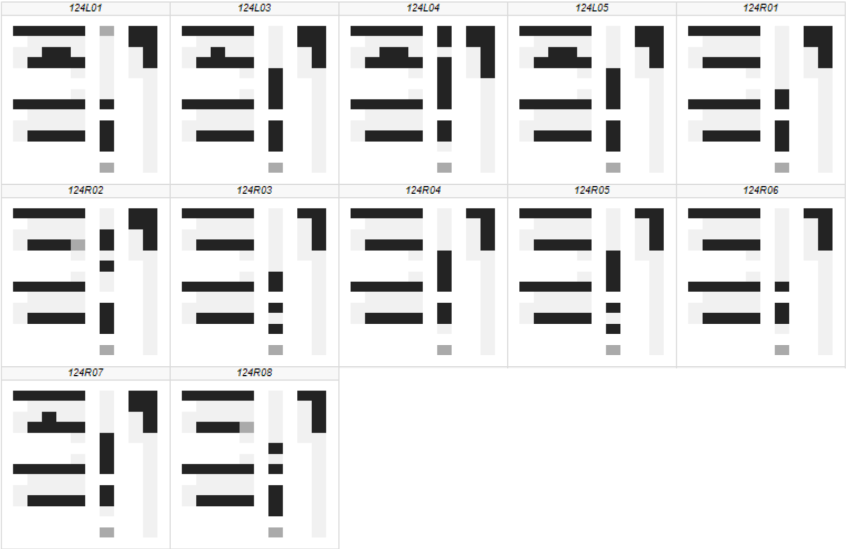

GD 21: Litters 201, 202, 203, 204

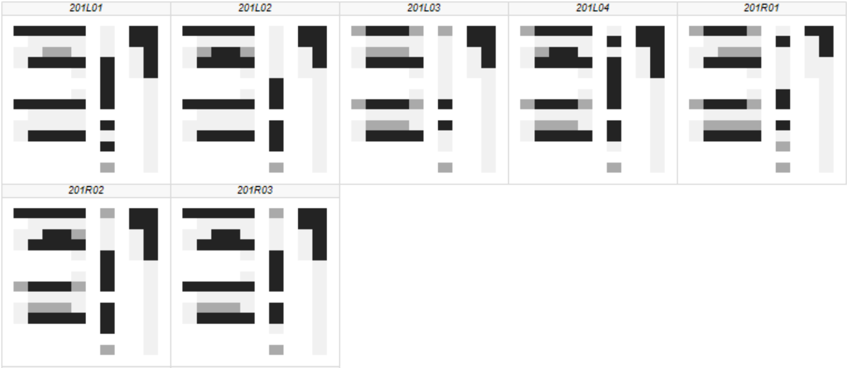

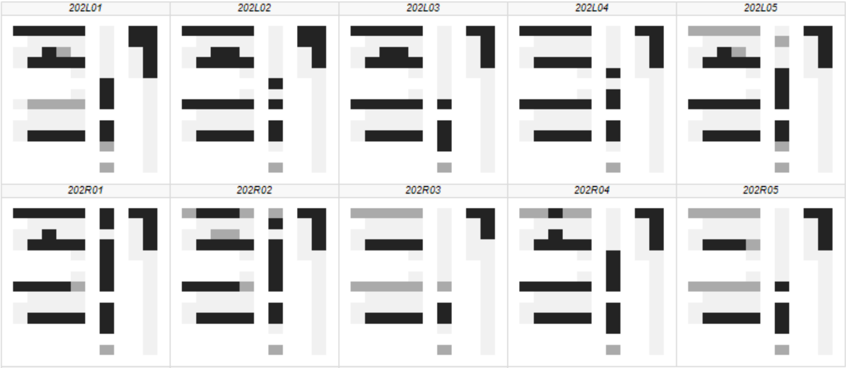

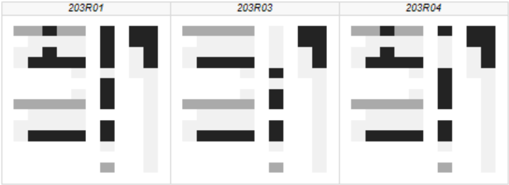

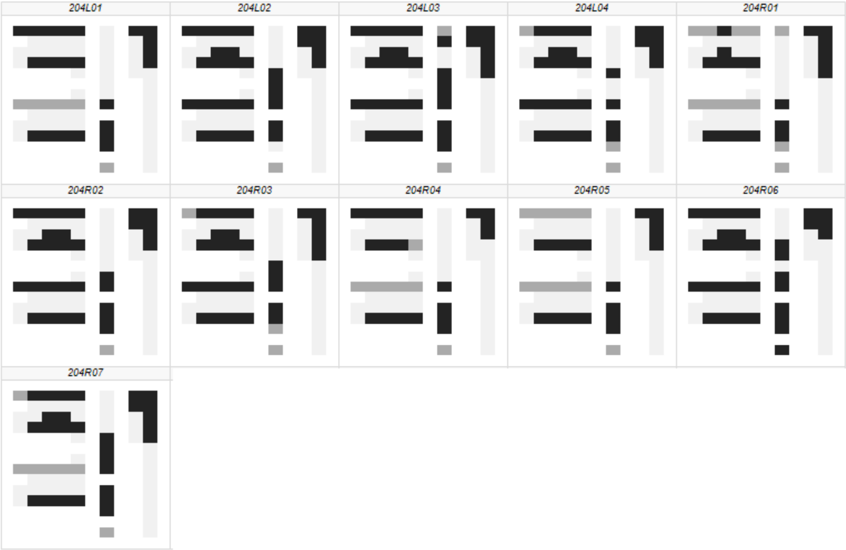

GD 21: Litters 205, 206, 207, 208

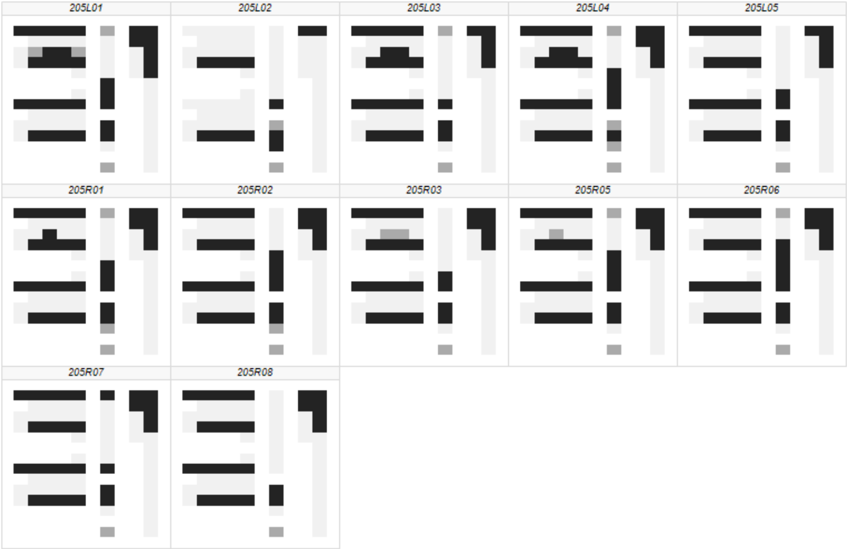

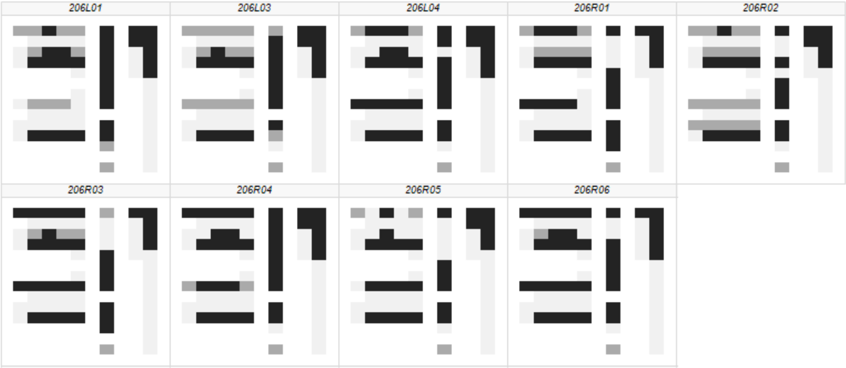

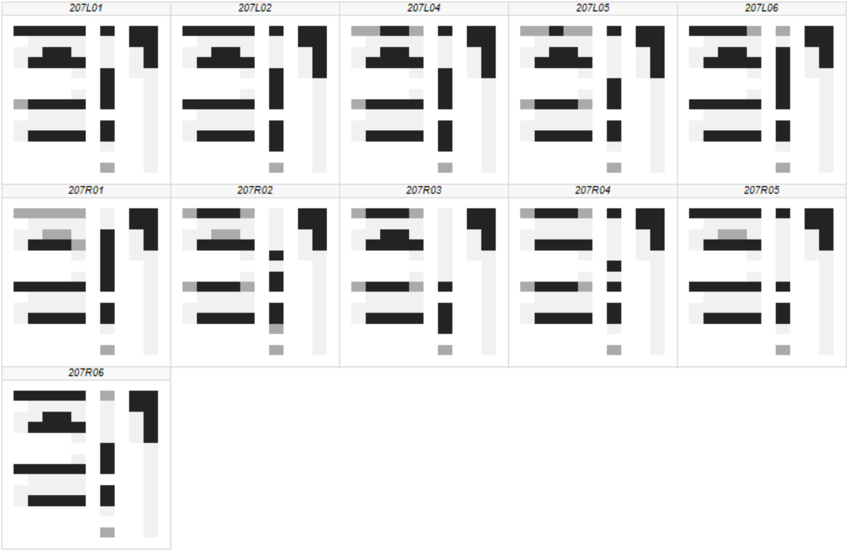

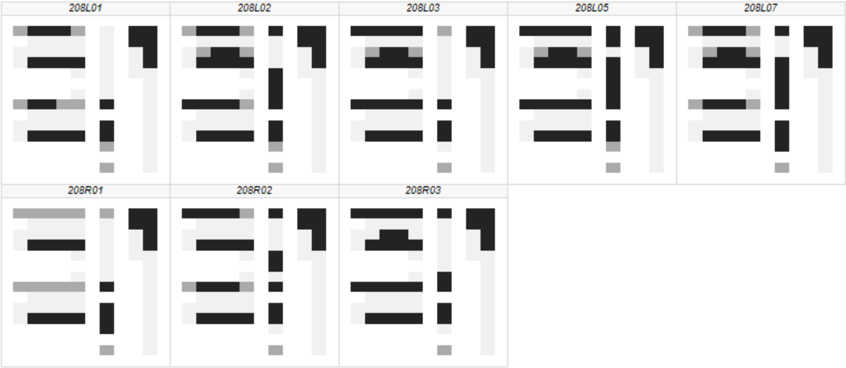

GD 21: Litters 209, 210, 211, 212

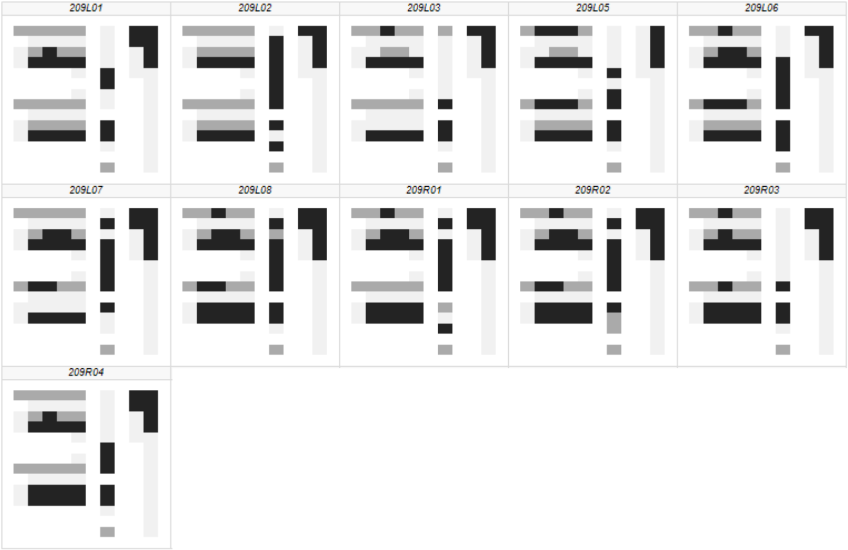

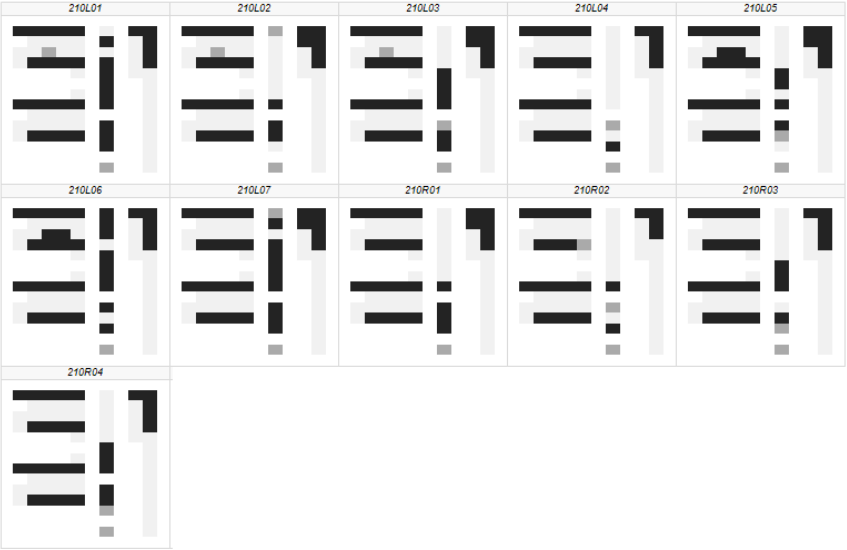

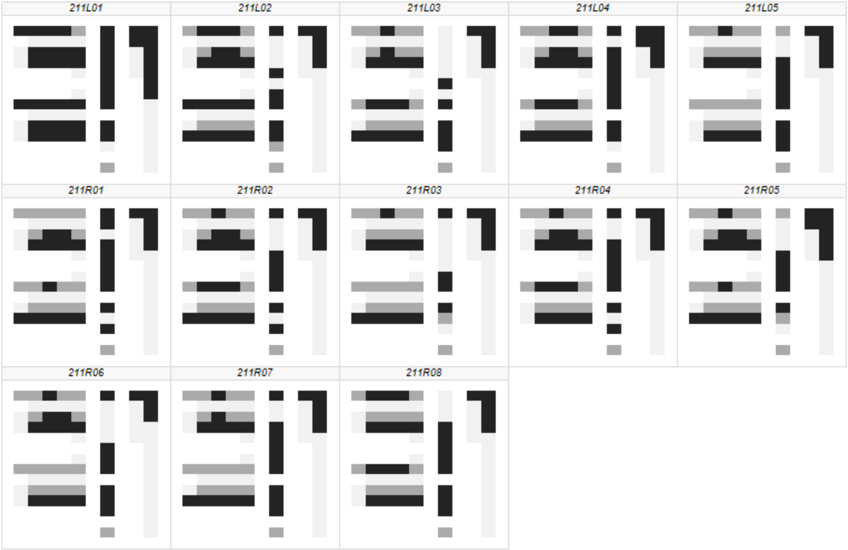

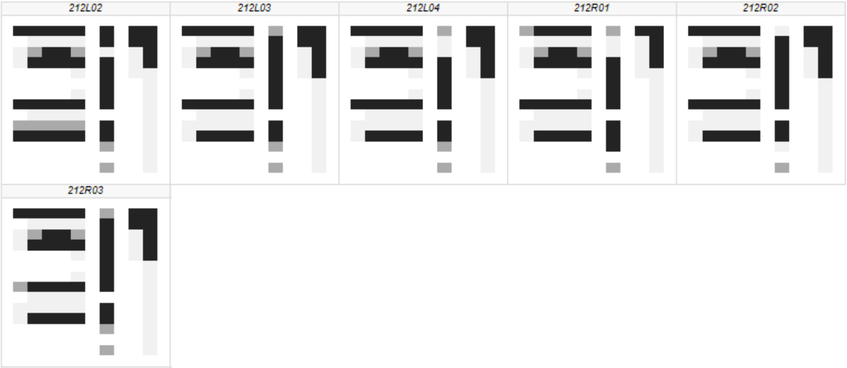

GD 21: Litters 214, 216, 217, 218

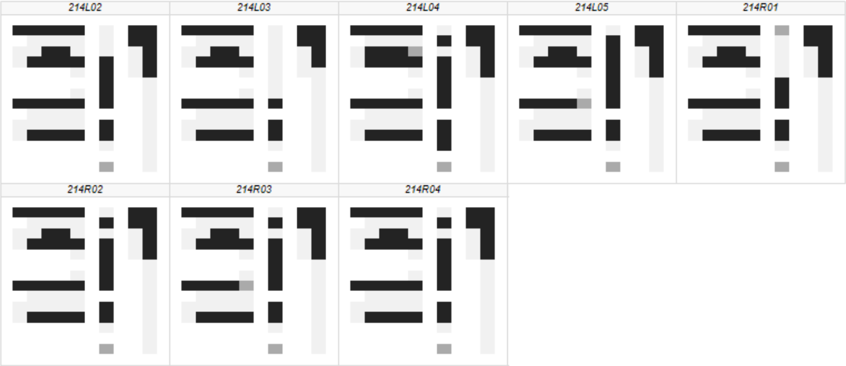

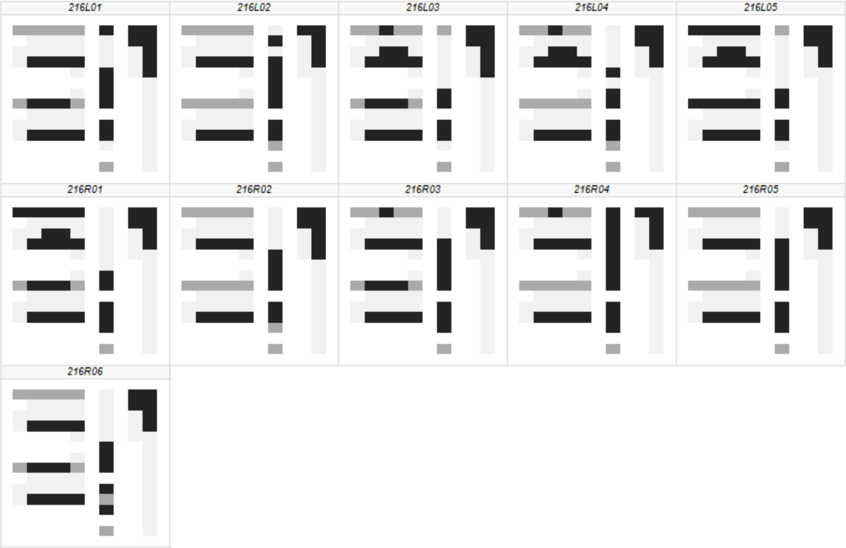

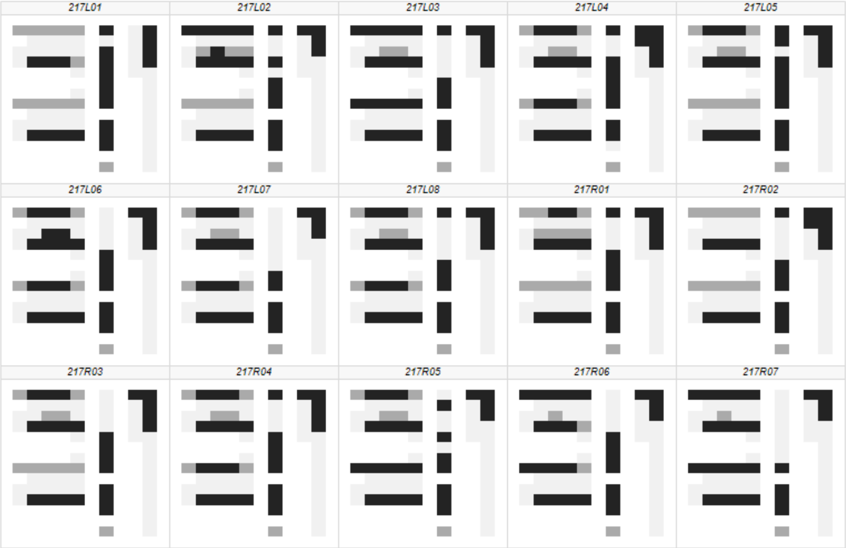

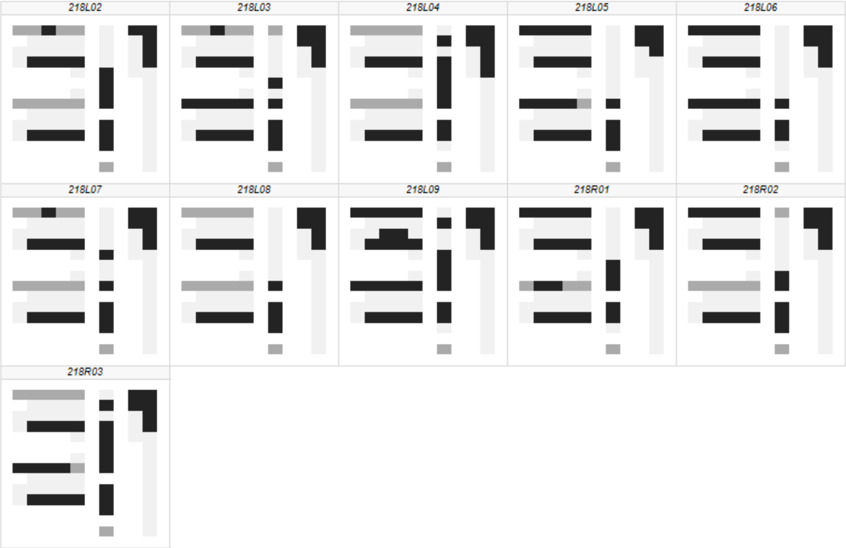

GD 21: Litters 219, 220, 221, 222

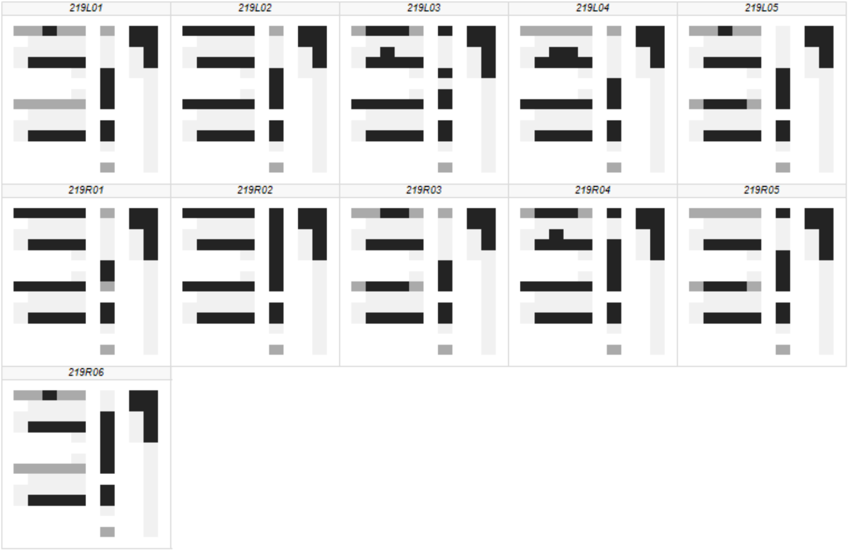

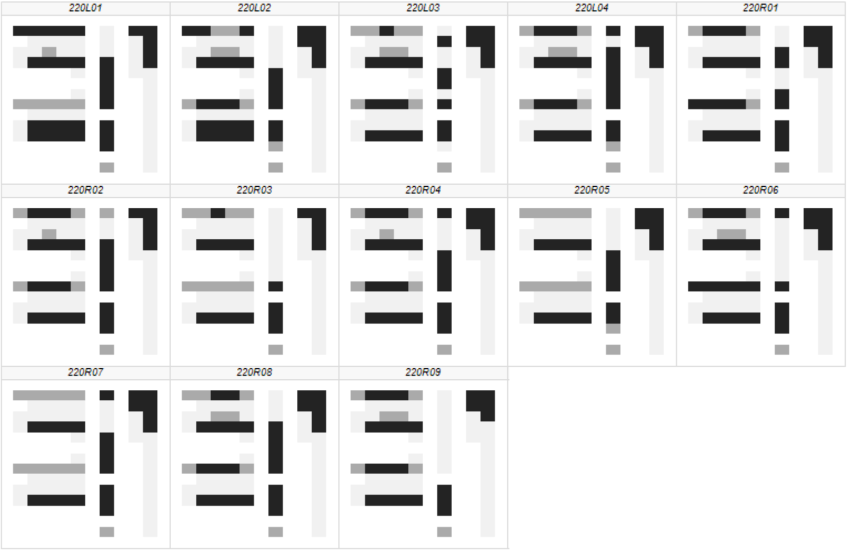

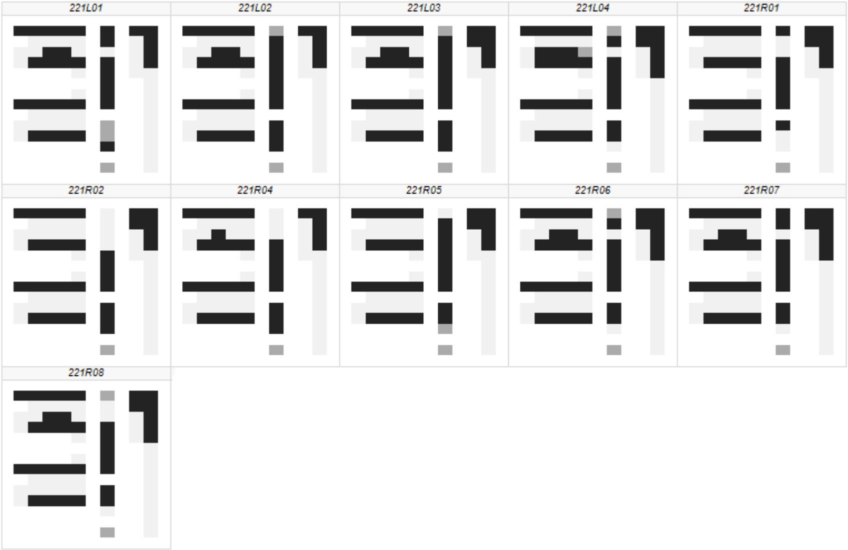

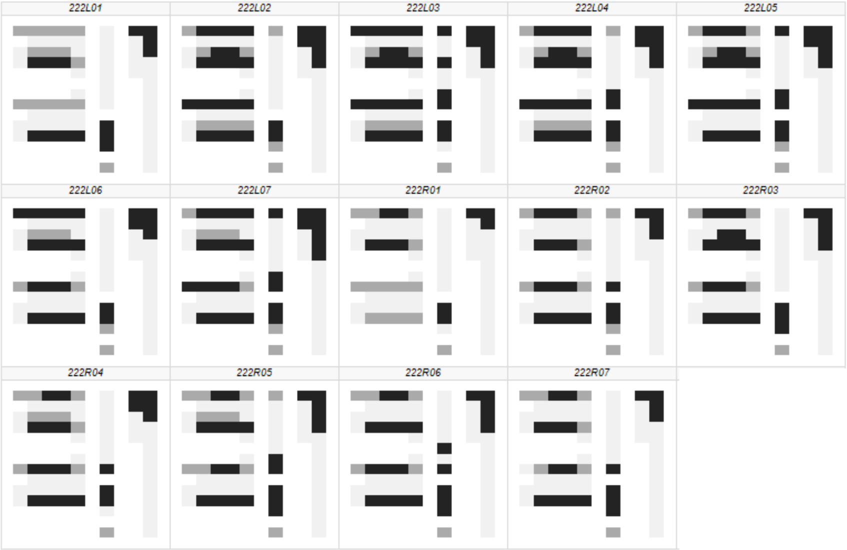

GD 21: Litters 223, 224

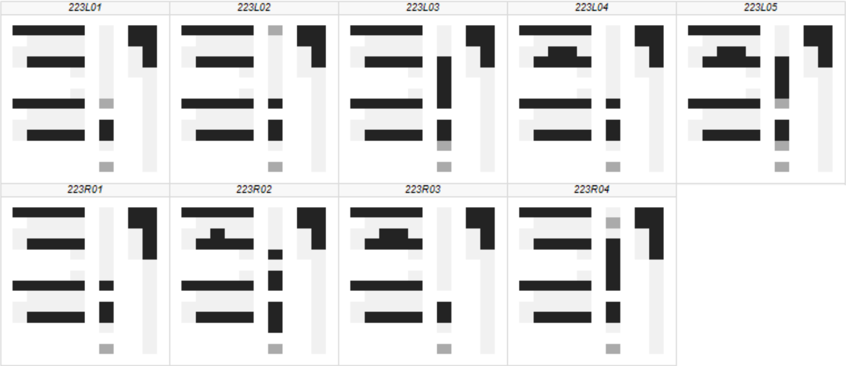

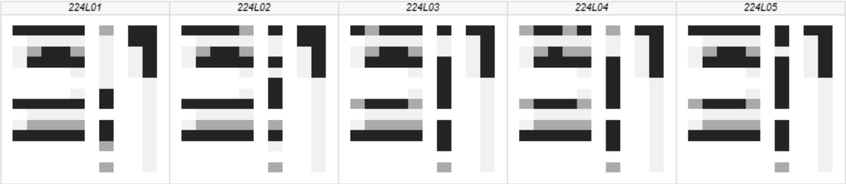

GD 22: Litters 301, 302, 303, 304

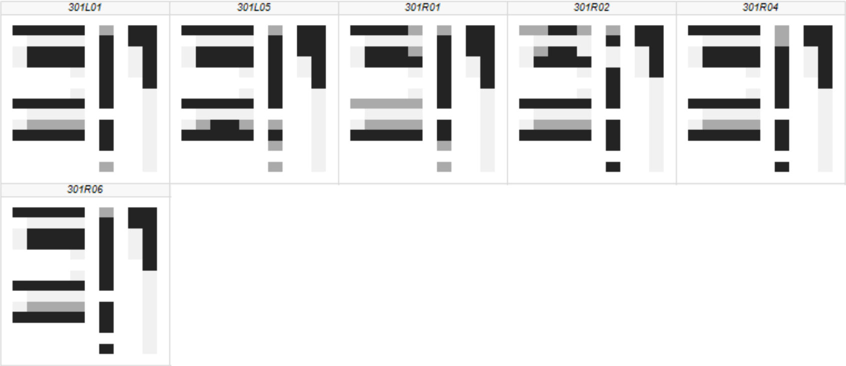

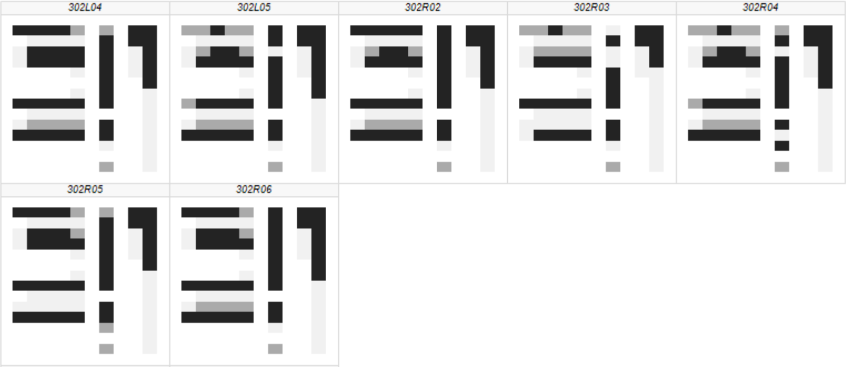

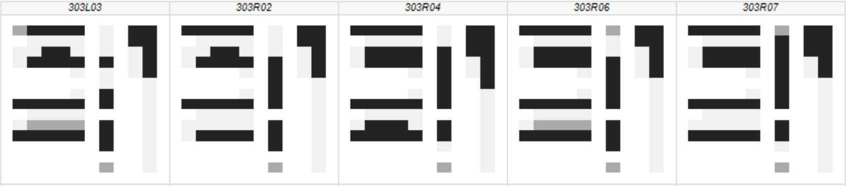

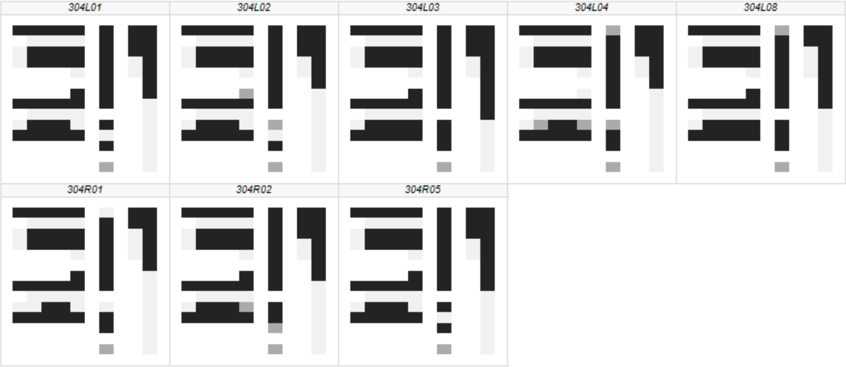

GD 22: Litters 305, 306, 307, 308

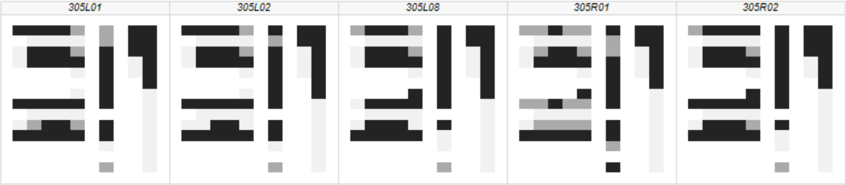

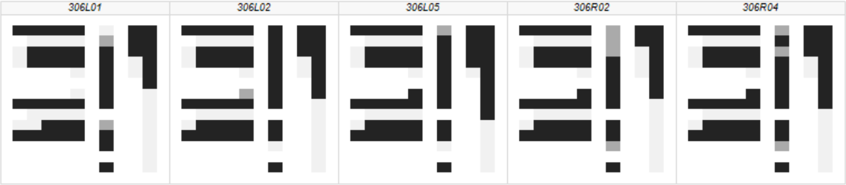

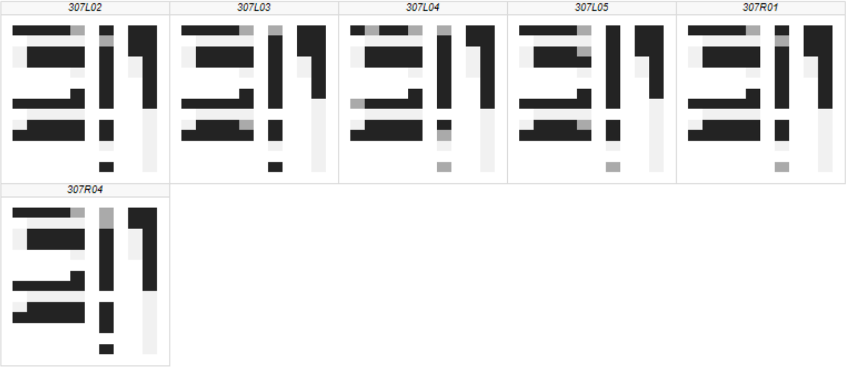

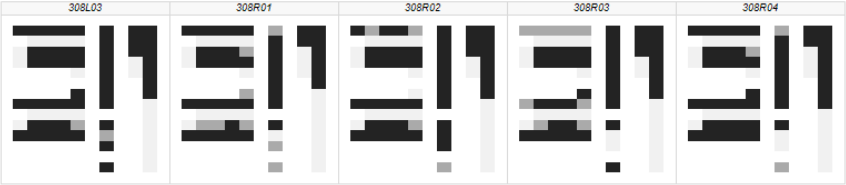

GD 22: Litters 309, 311, 312, 313

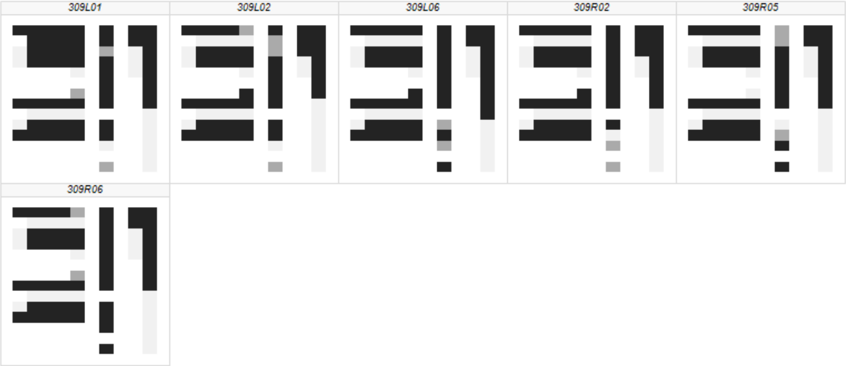

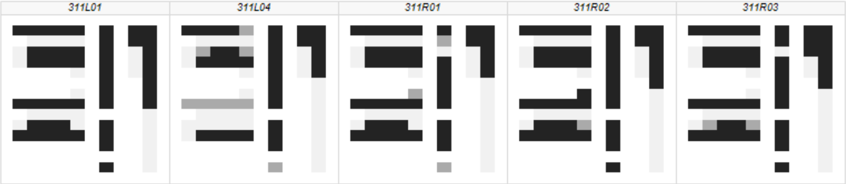

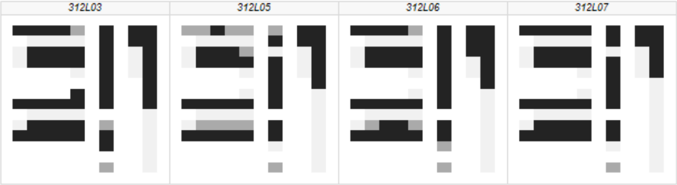

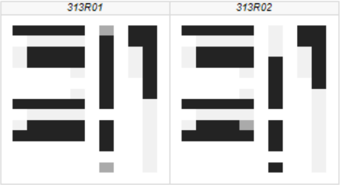

GD 22: Litters 314, 316, 317, 318

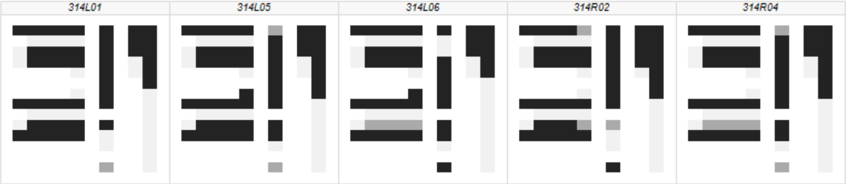

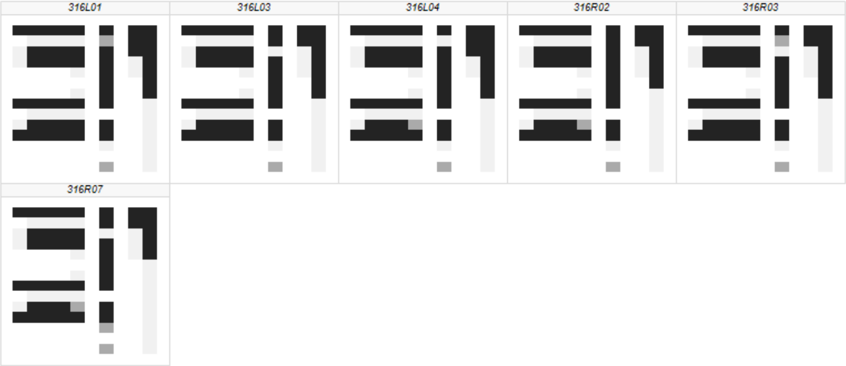

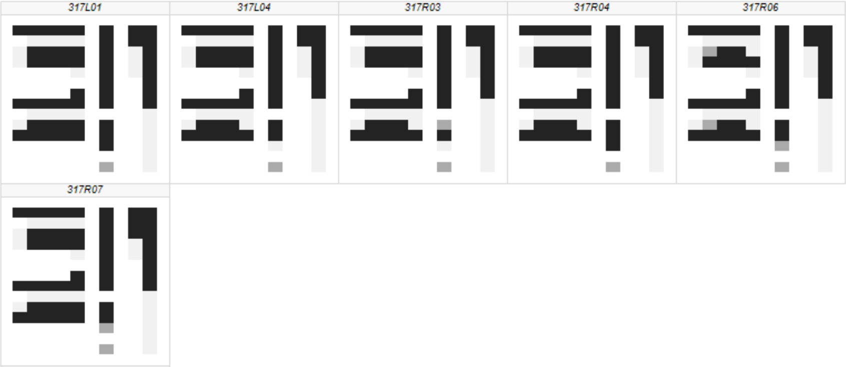

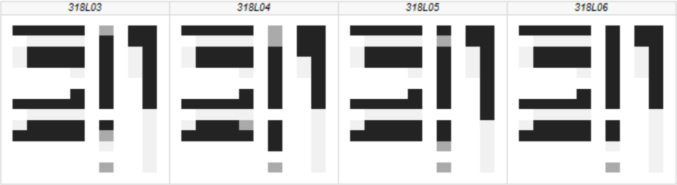

GD 22: Litters 319, 321, 322, 324

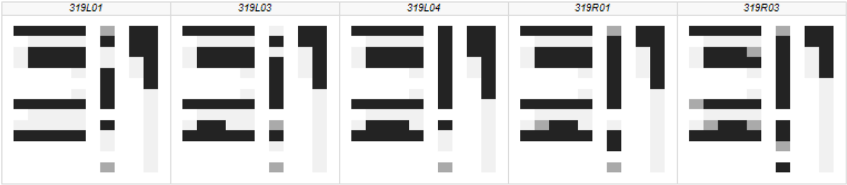

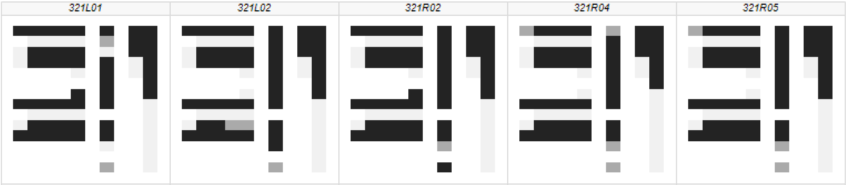

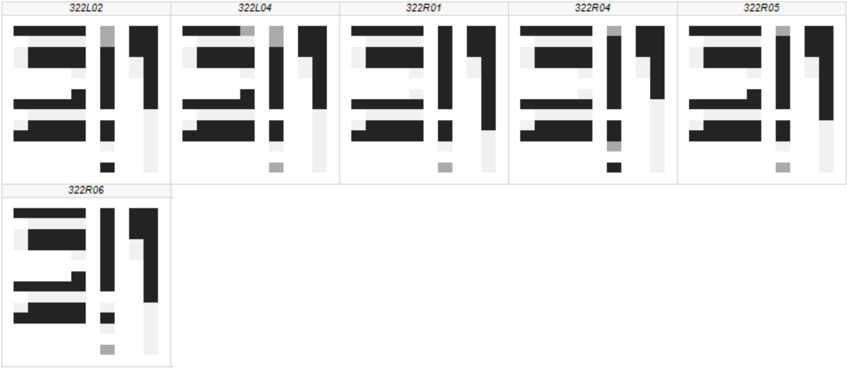

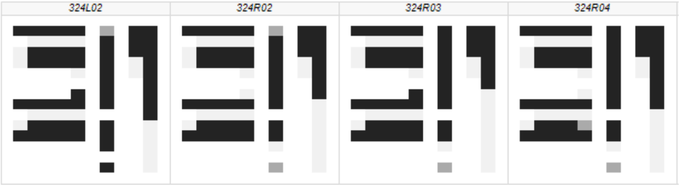

GD 22: Litters 401, 402, 403, 405

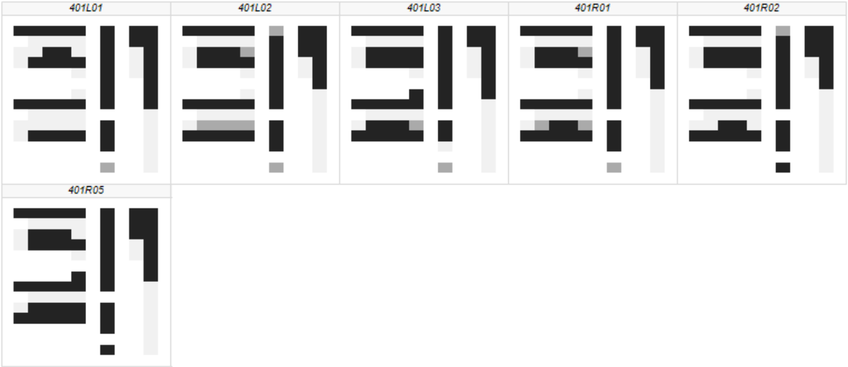

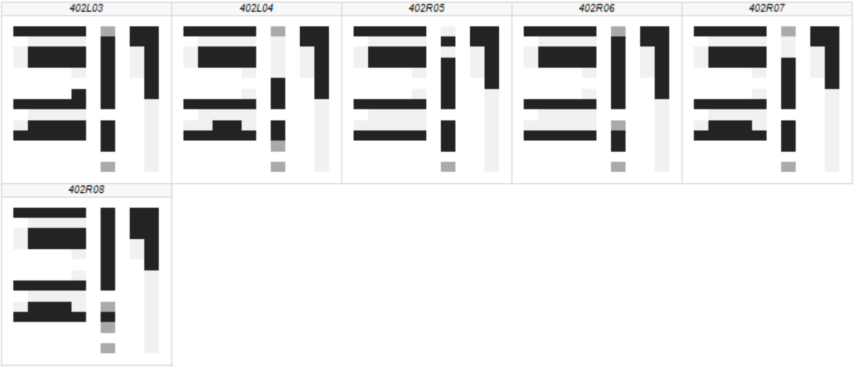

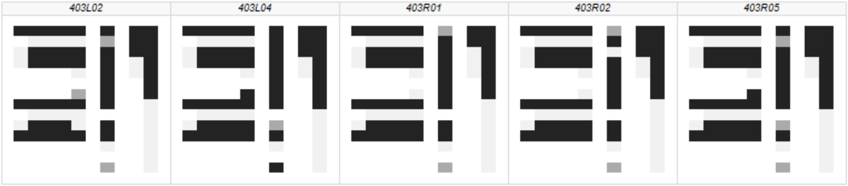

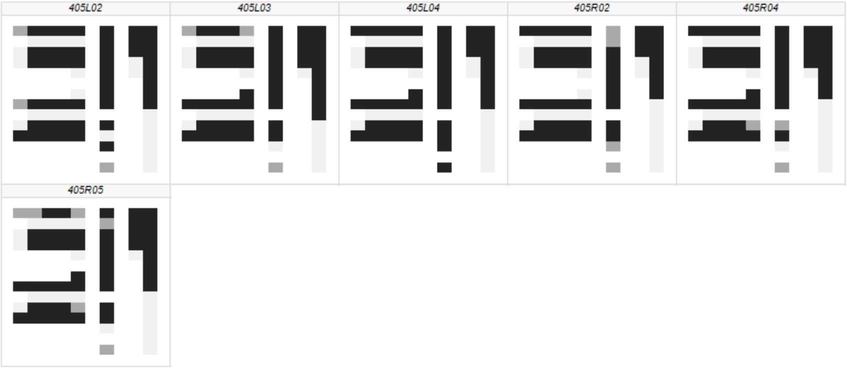

GD 22: Litters 406, 407, 408, 409

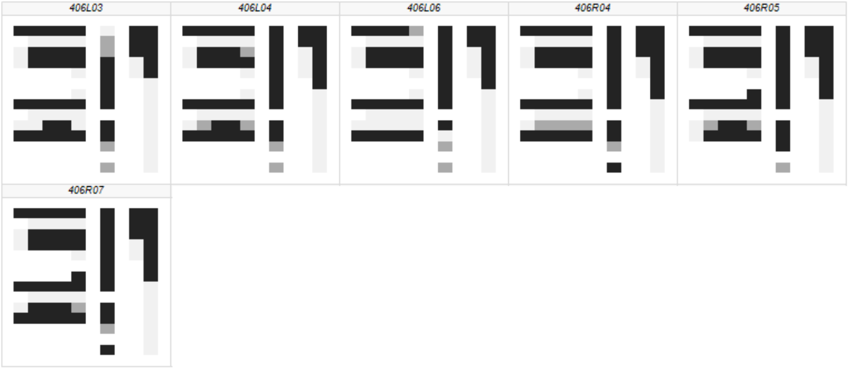

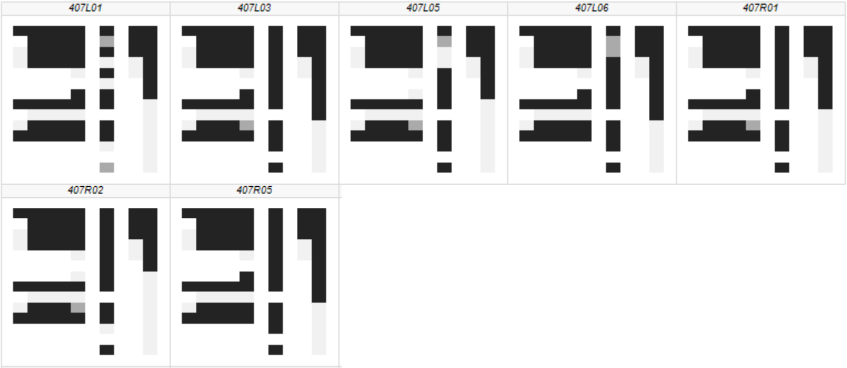

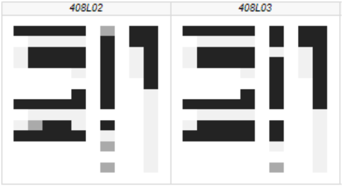

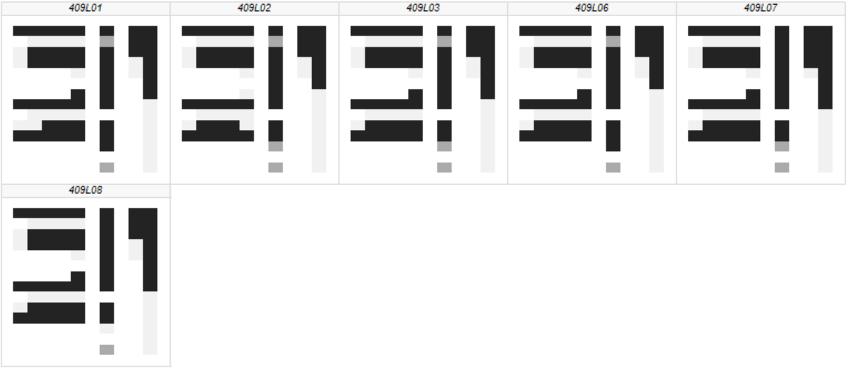

GD 22: Litters 410, 413, 414, 415

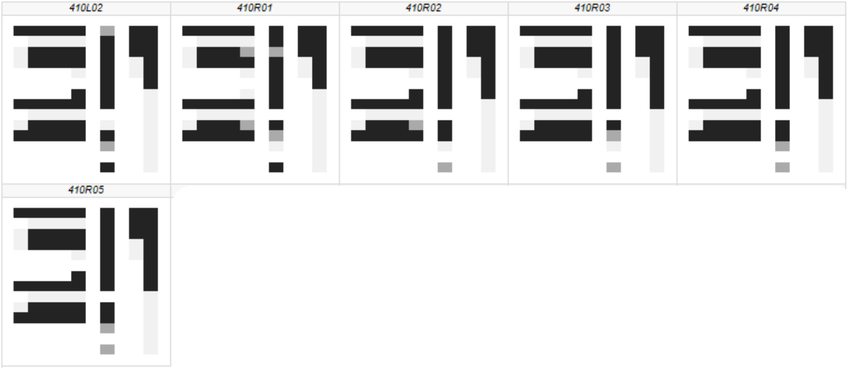

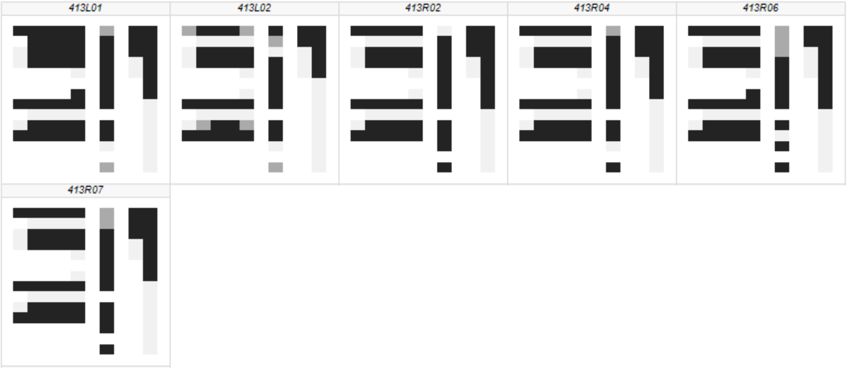

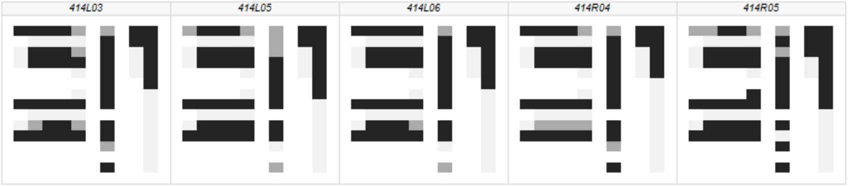

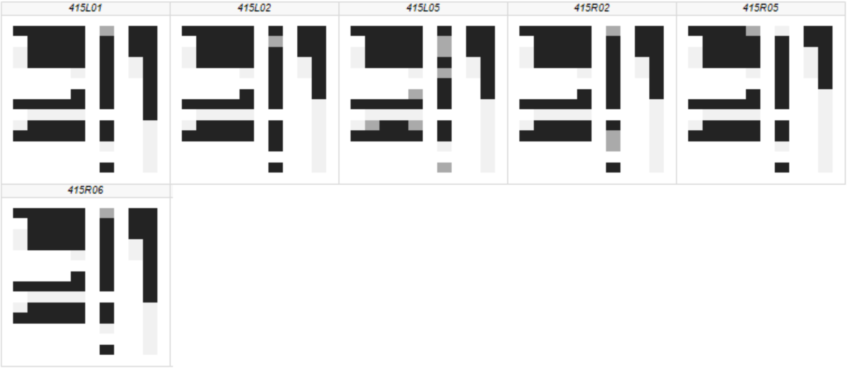

GD 22: Litters 416, 417, 418, 419

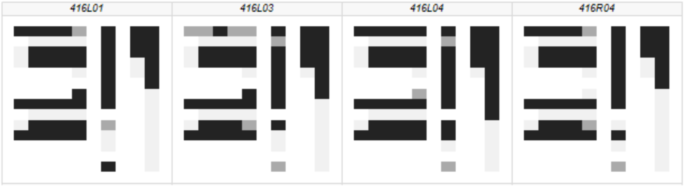

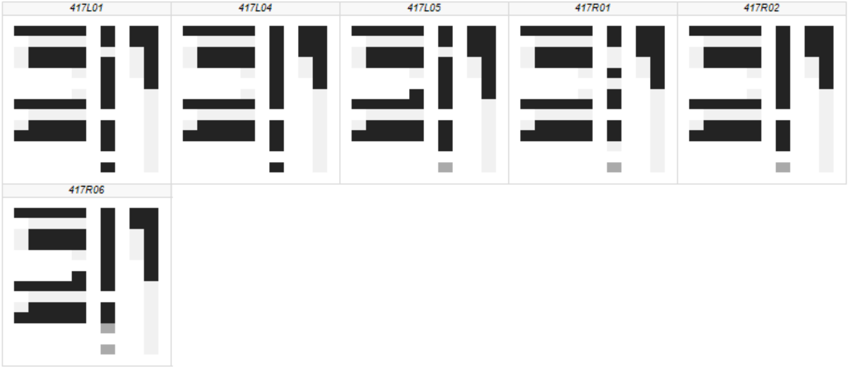

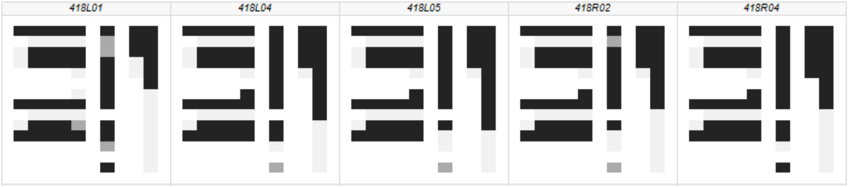

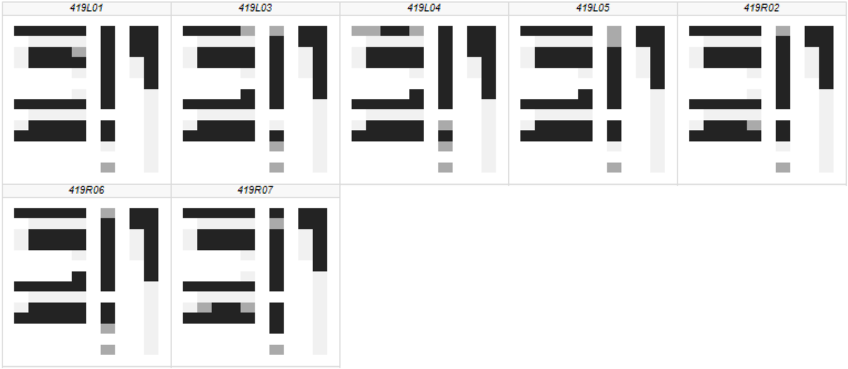

GD 22: Litters 420, 421, 422, 423

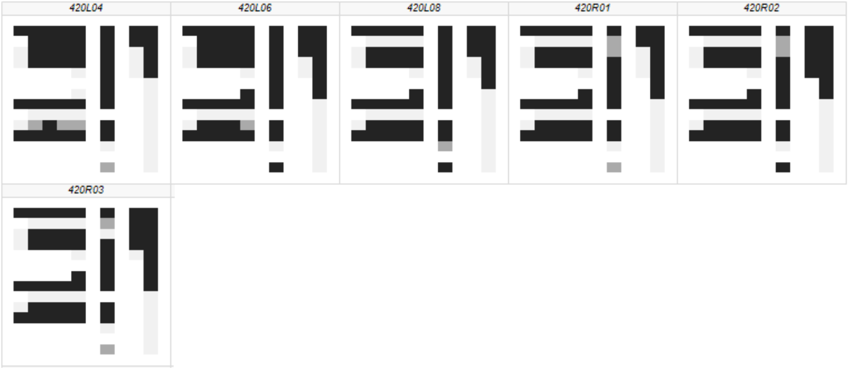

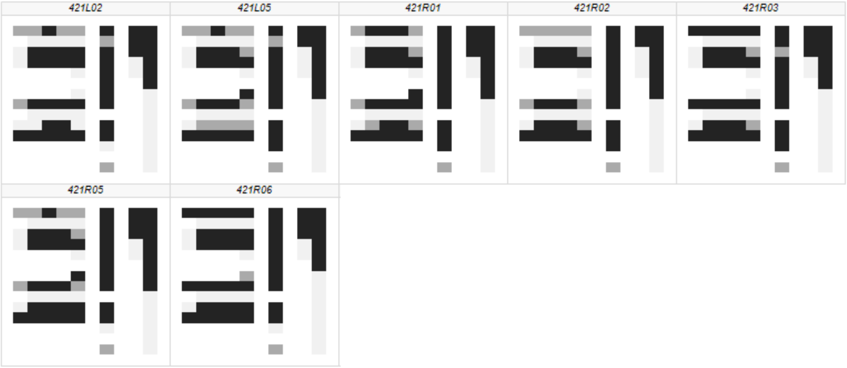

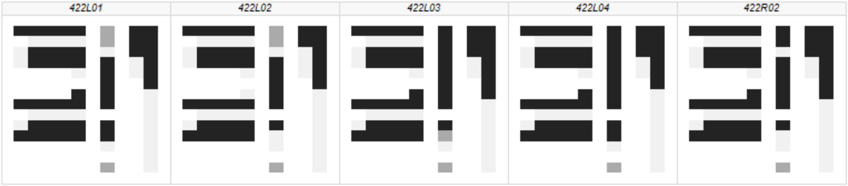

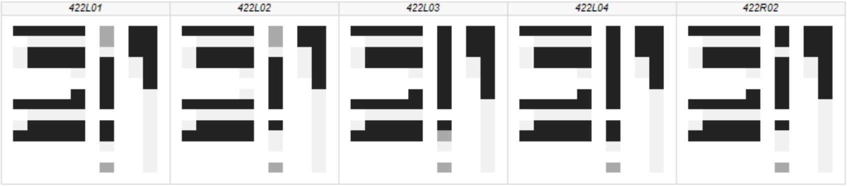

GD 22: Litters 424

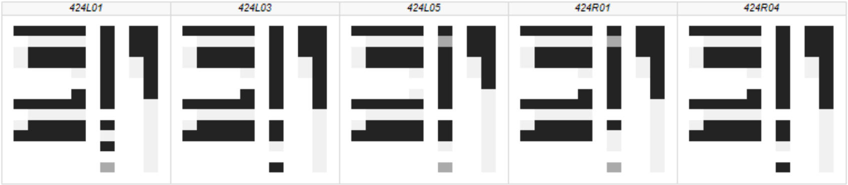

GD 23: Litters 503, 504, 505, 506

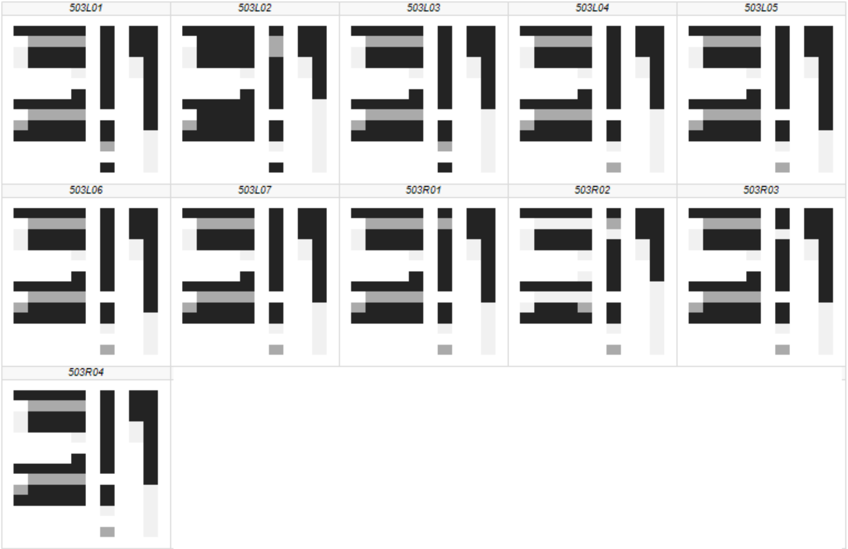

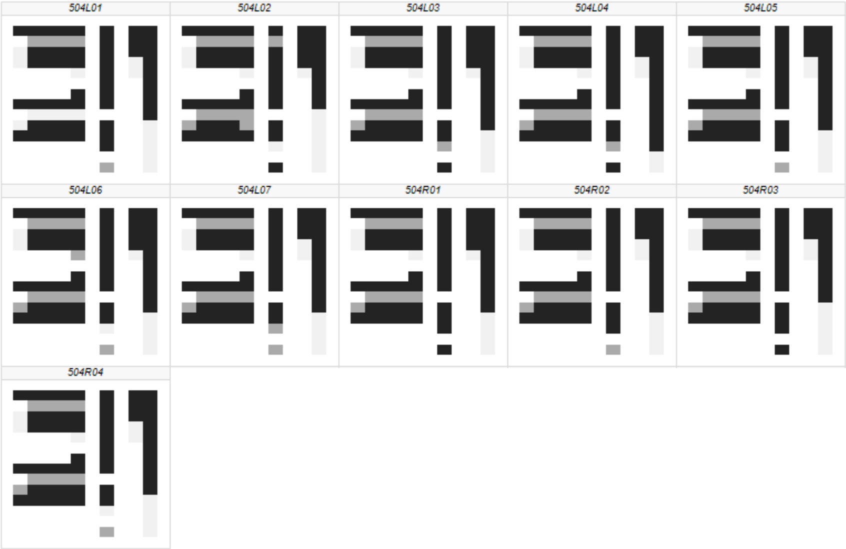

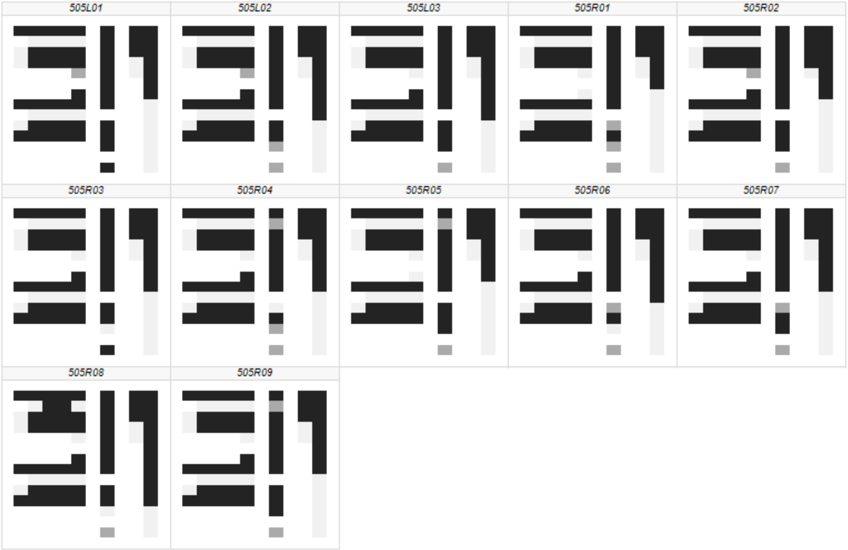

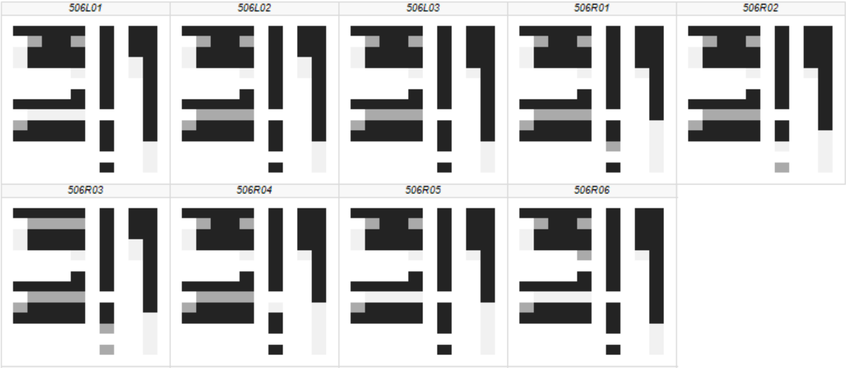

GD 23: Litters 507, 510, 511, 513

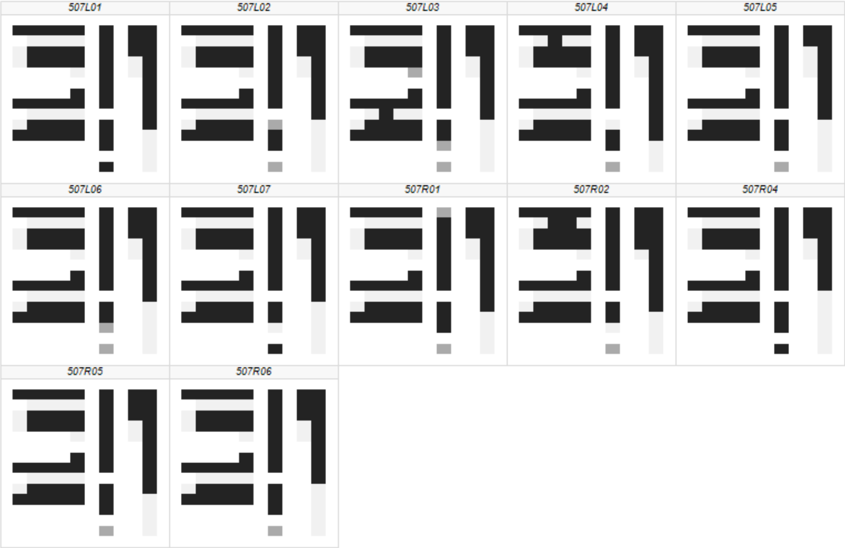

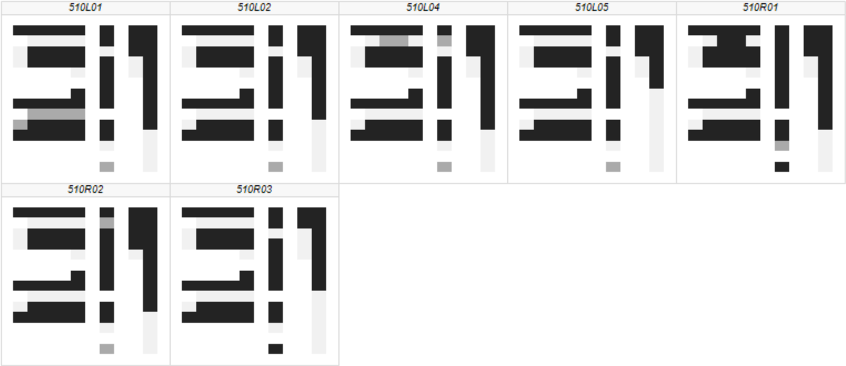

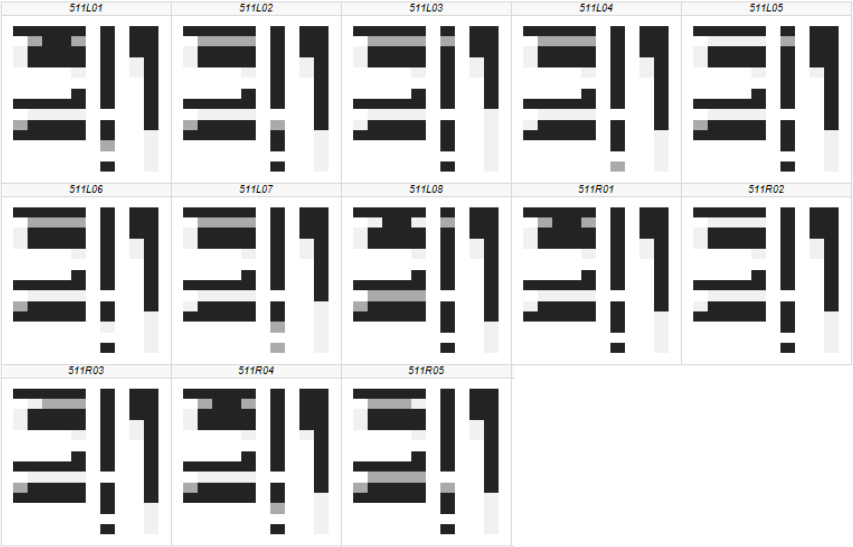

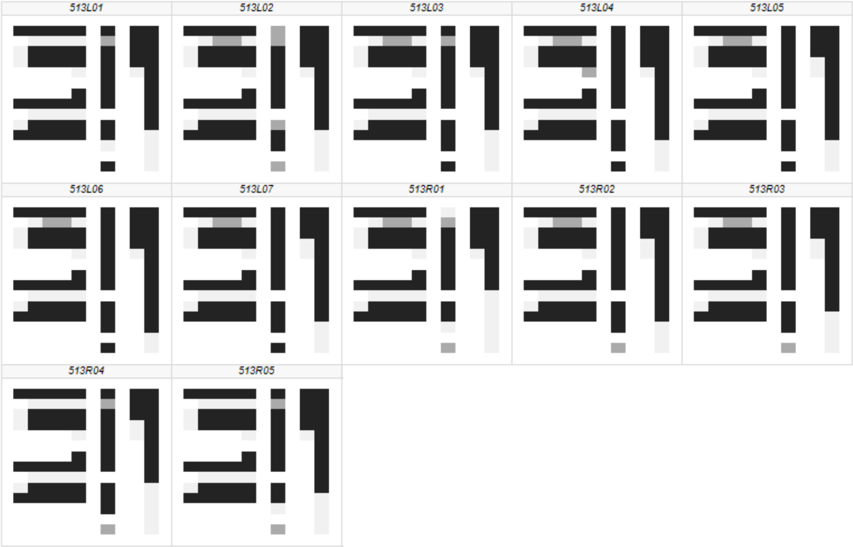

GD 23: Litters 514, 515, 516, 517

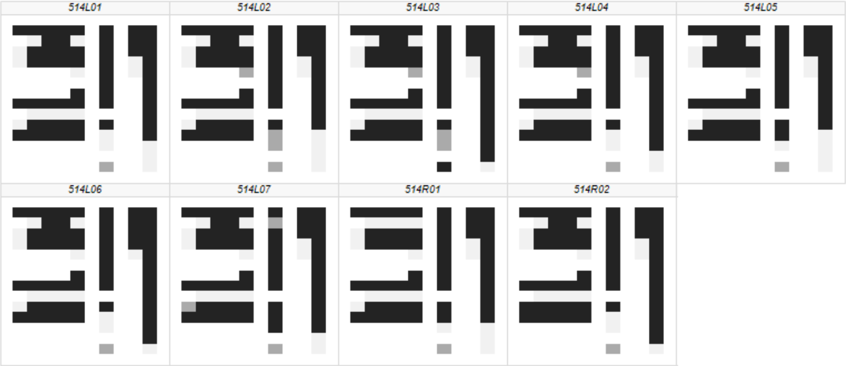

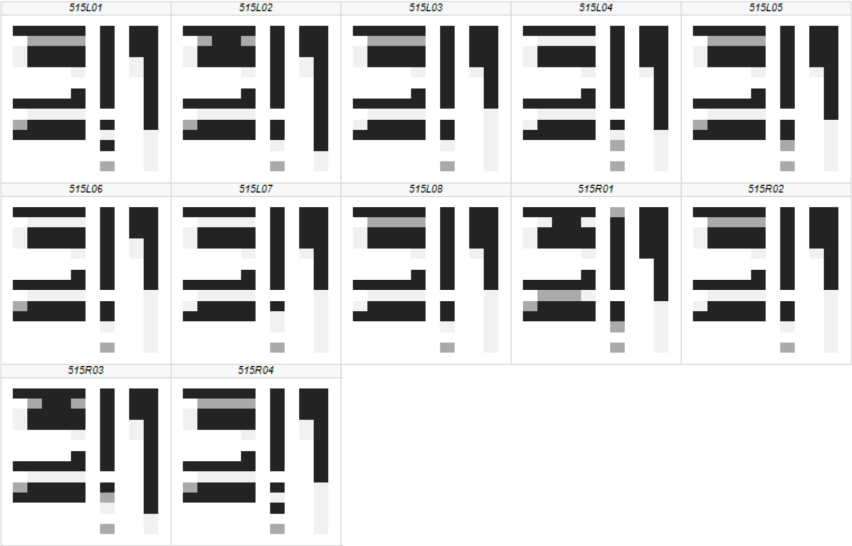

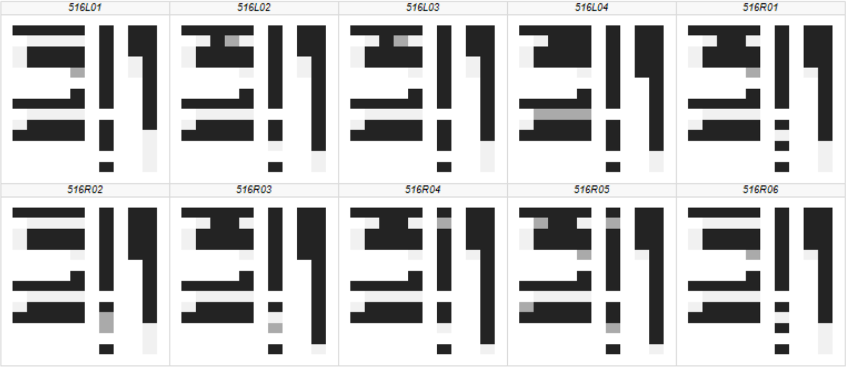

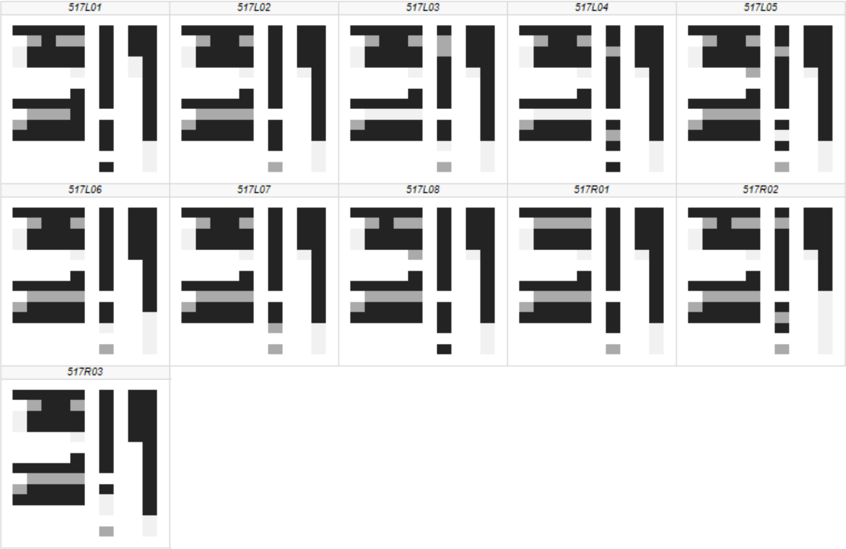

GD 23: Litters 518, 520, 521, 522

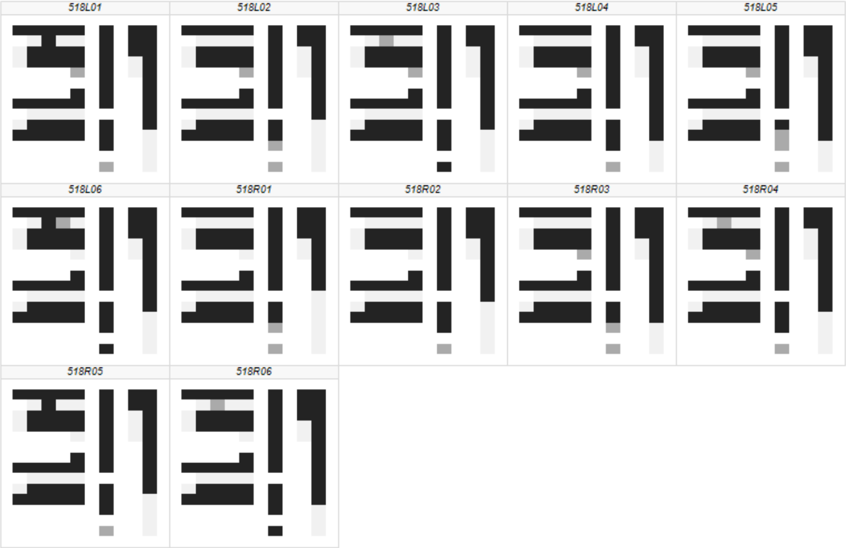

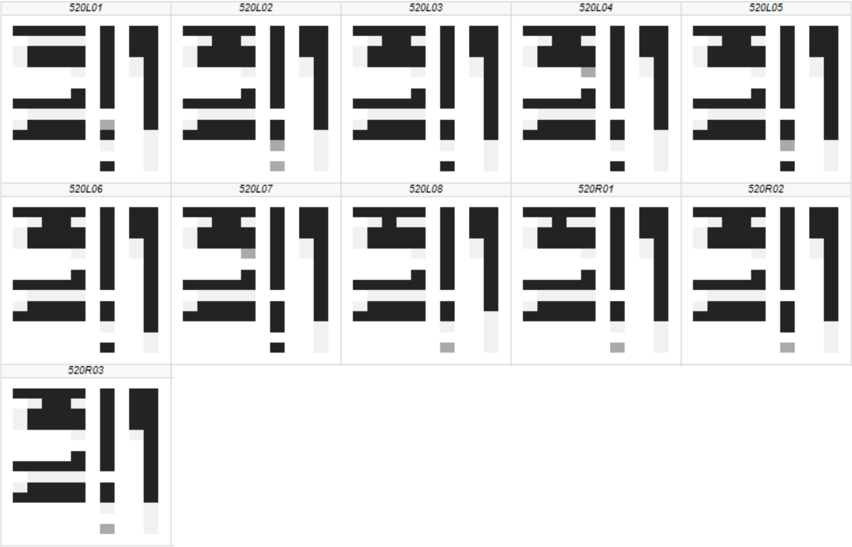

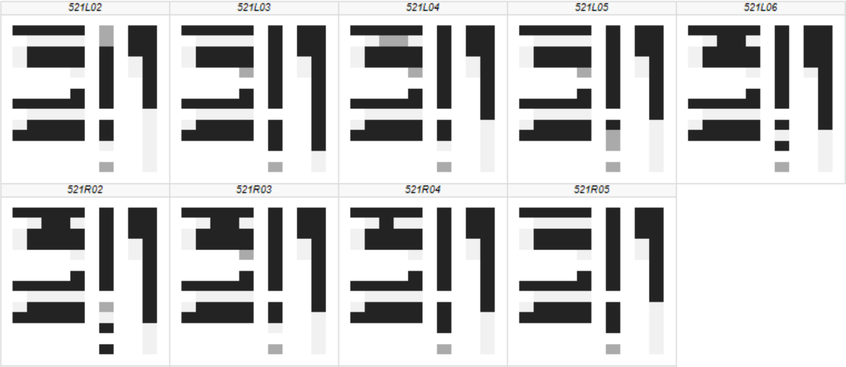

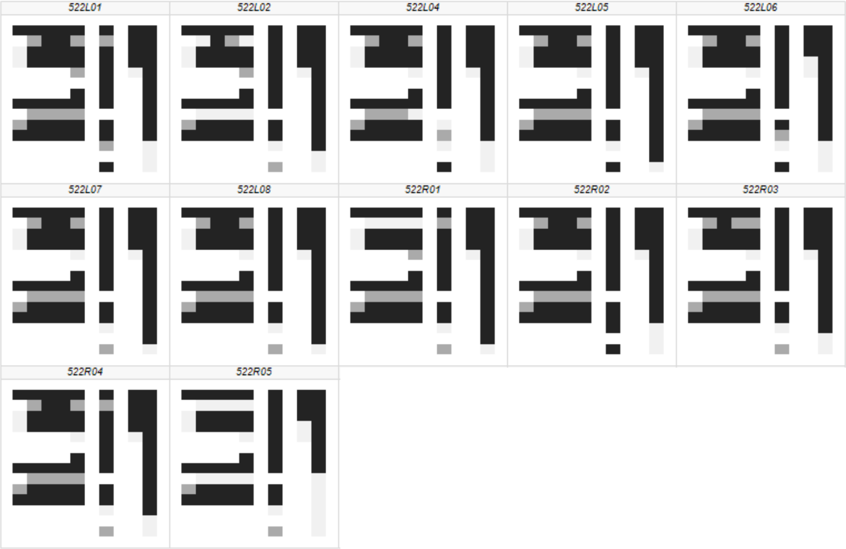

GD 23: Litters 523, 524

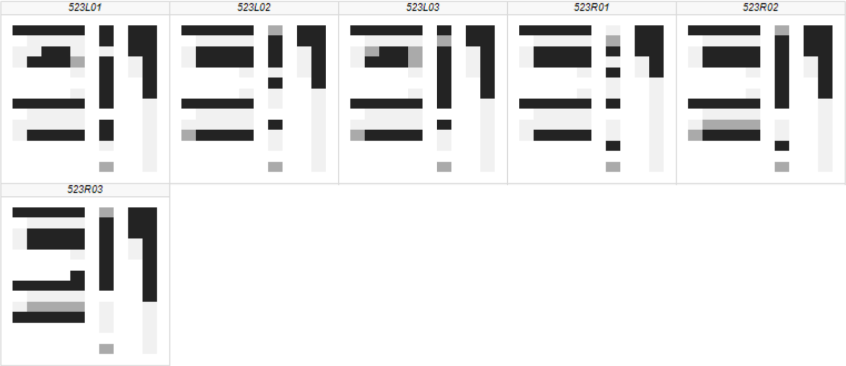

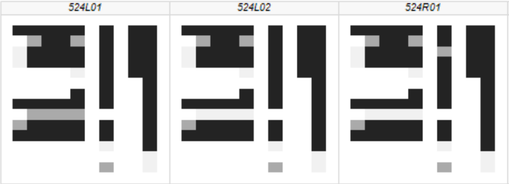

GD 23: Litters 601, 602, 603, 604

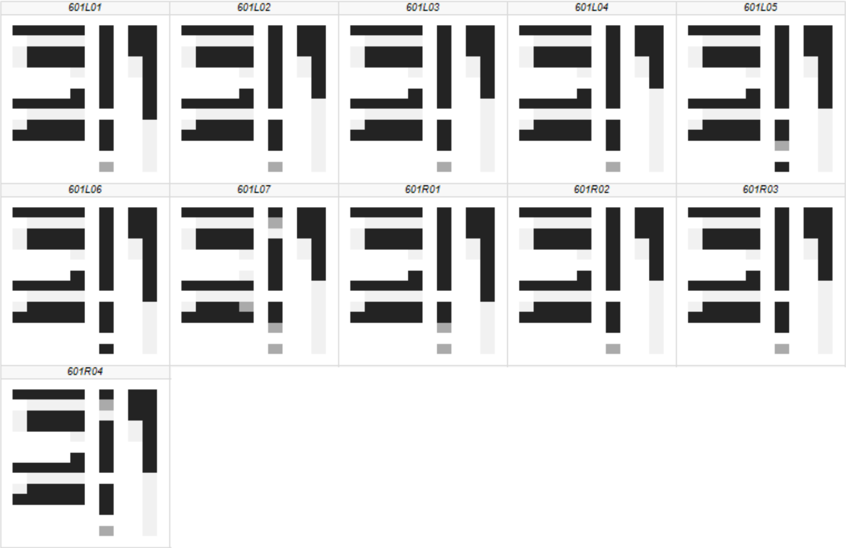

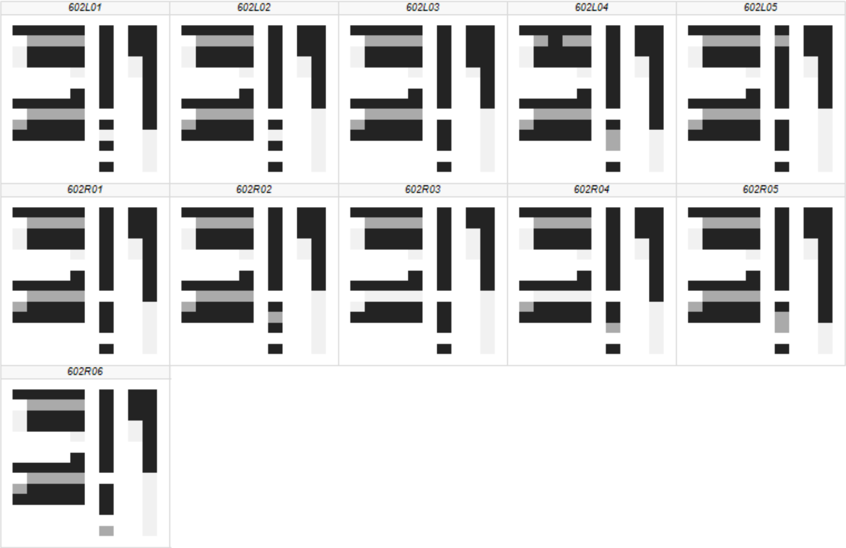

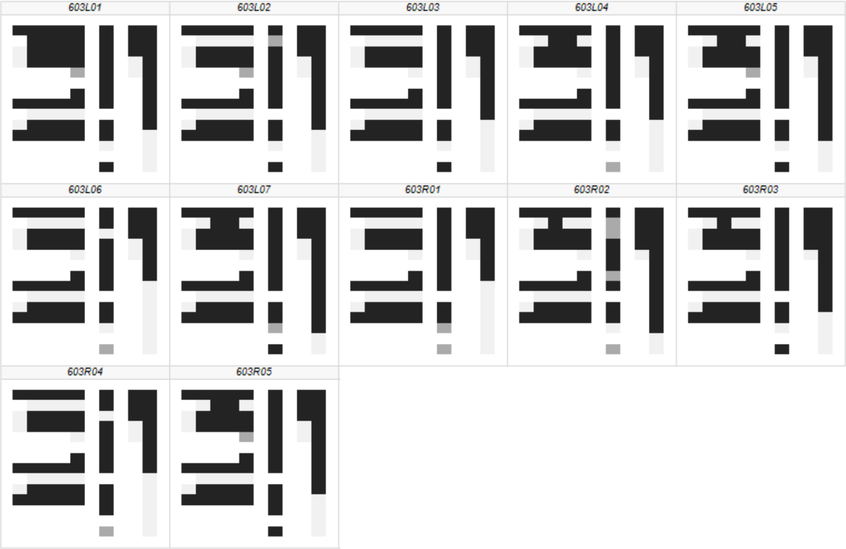

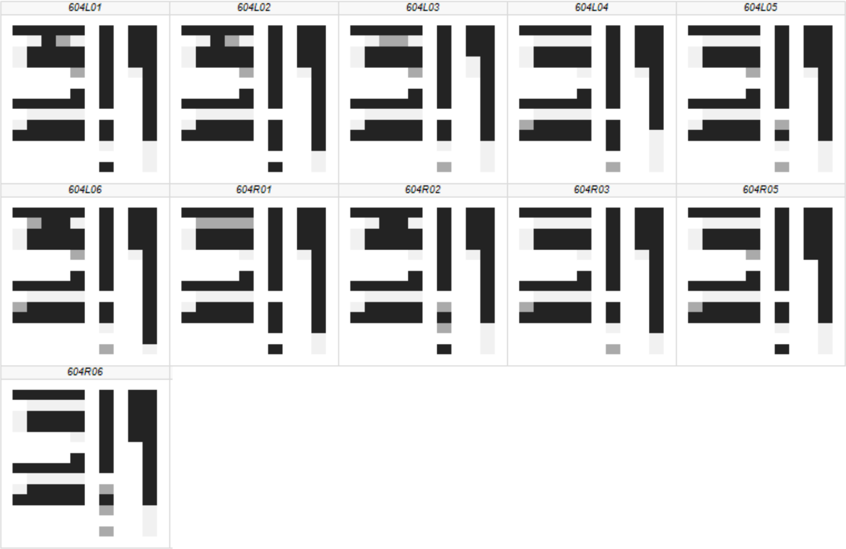

GD 23: Litters 605, 606, 608, 609

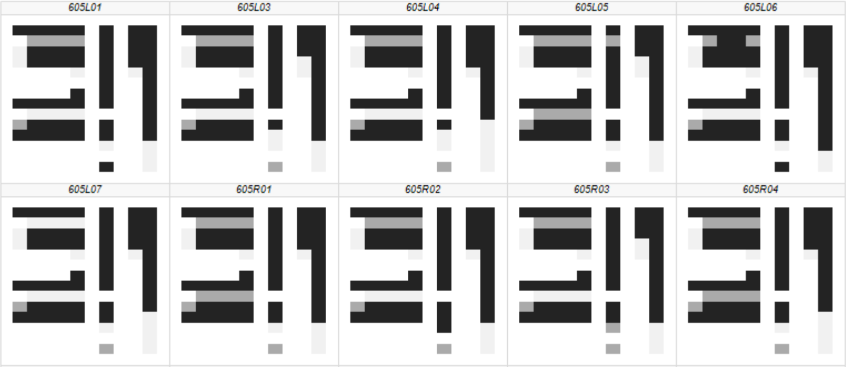

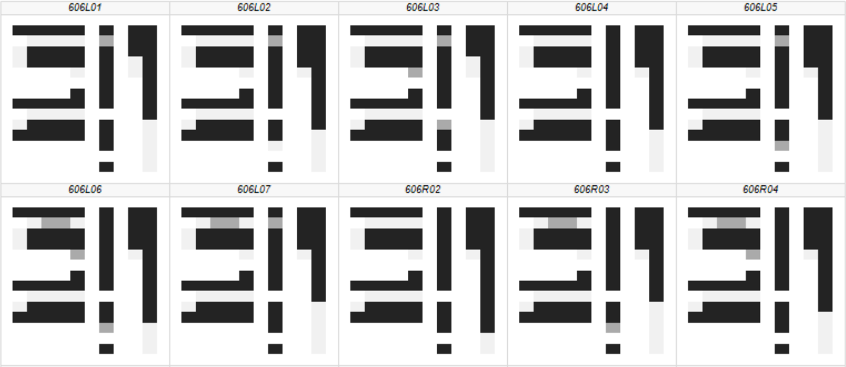

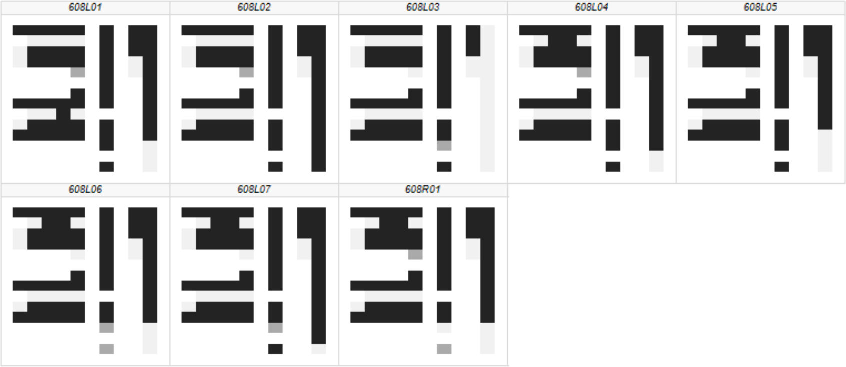

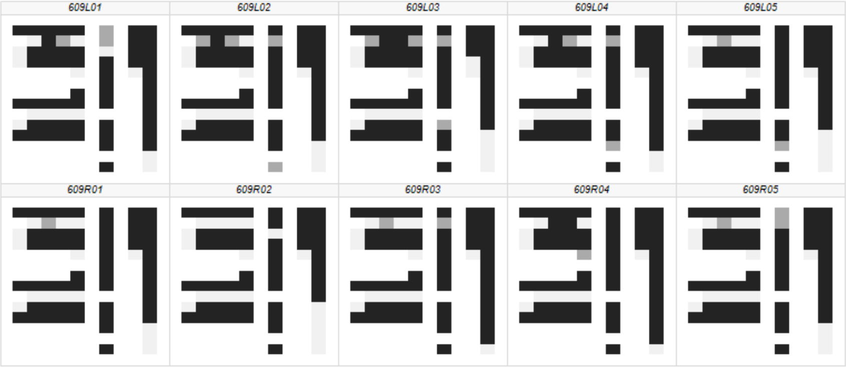

GD 23: Litters 611, 612, 613, 614

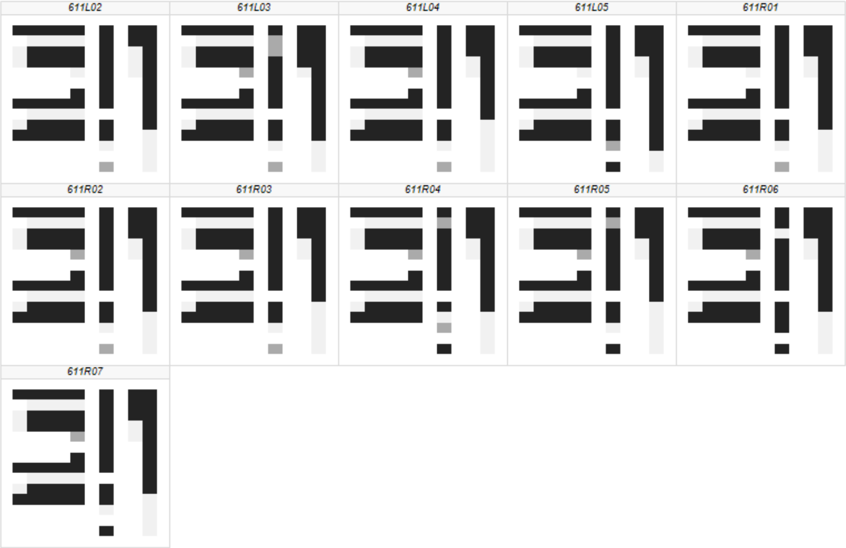

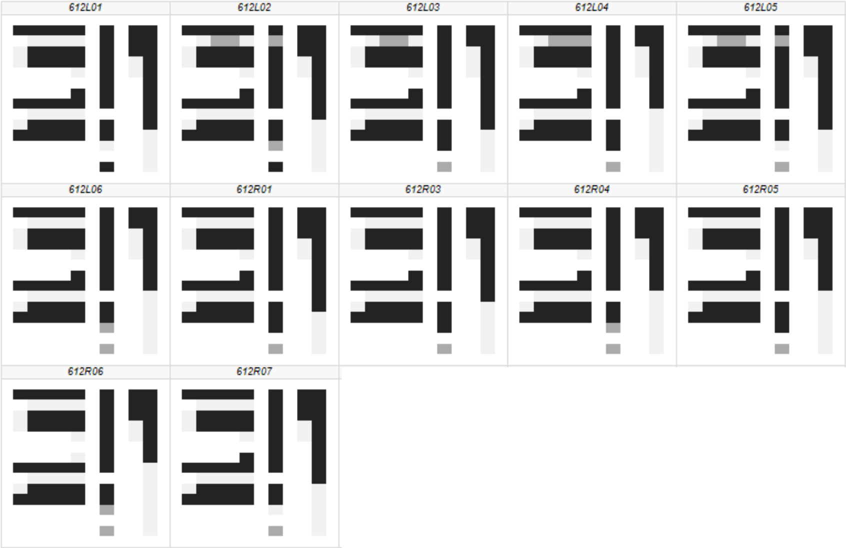

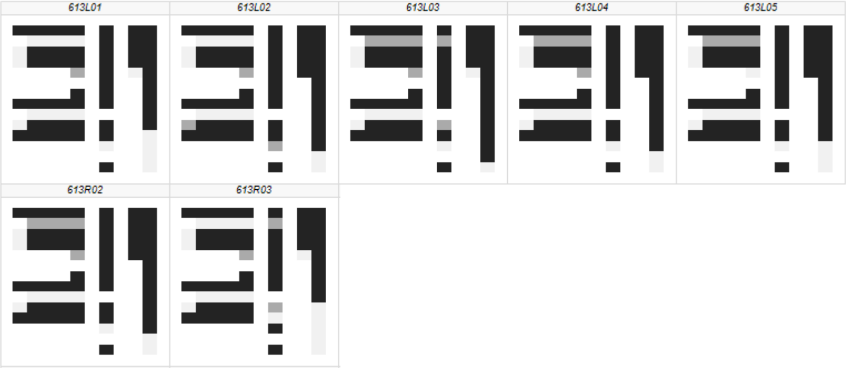

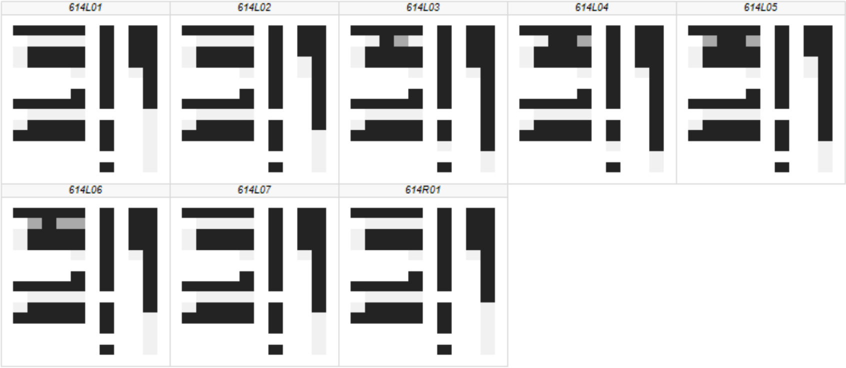

GD 23: Litters 615, 617, 618, 619

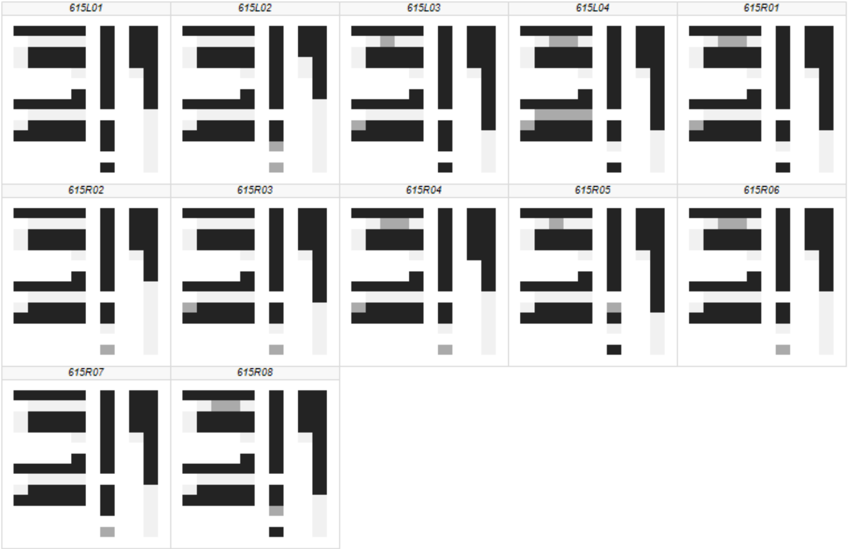

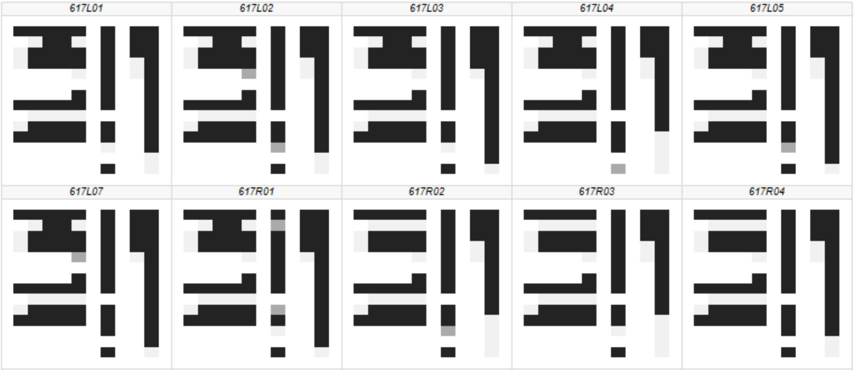

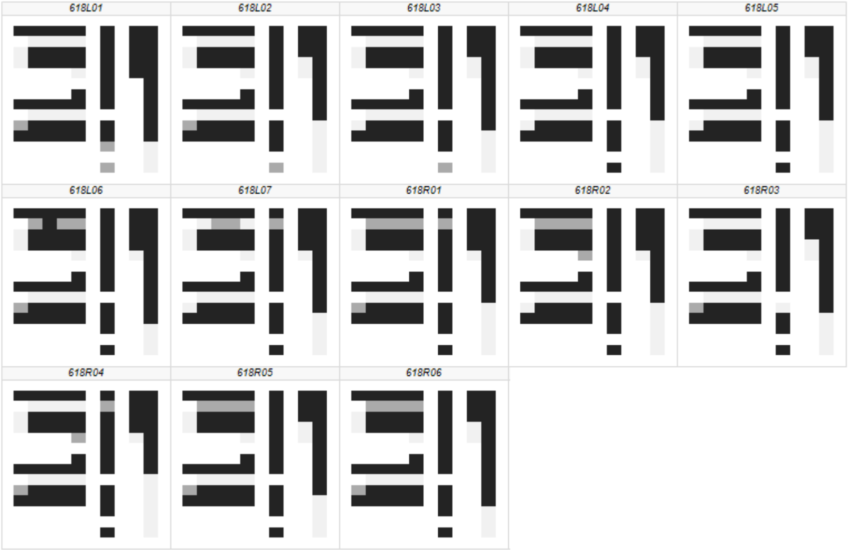

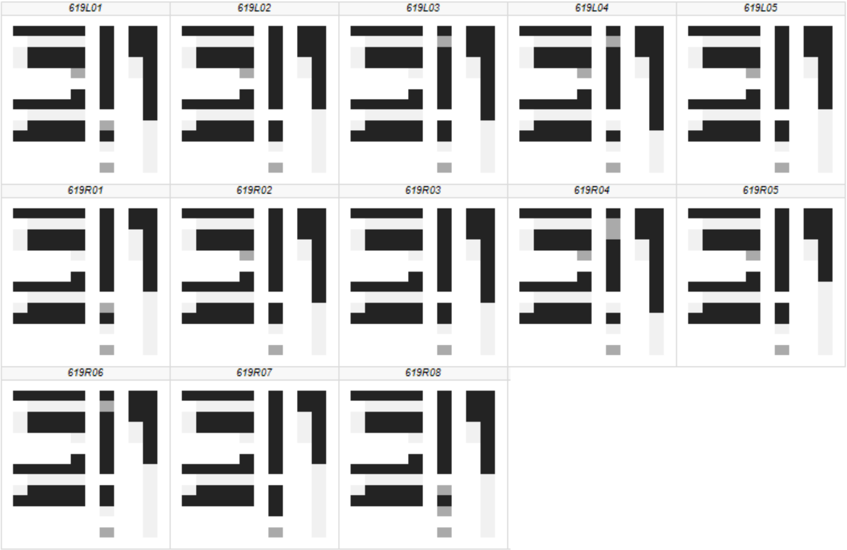

GD 23: Litters 621, 622, 624

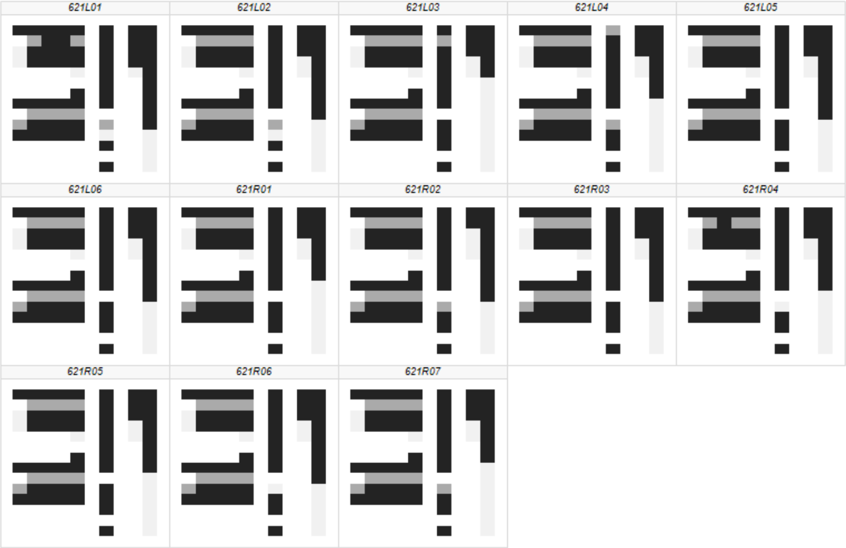

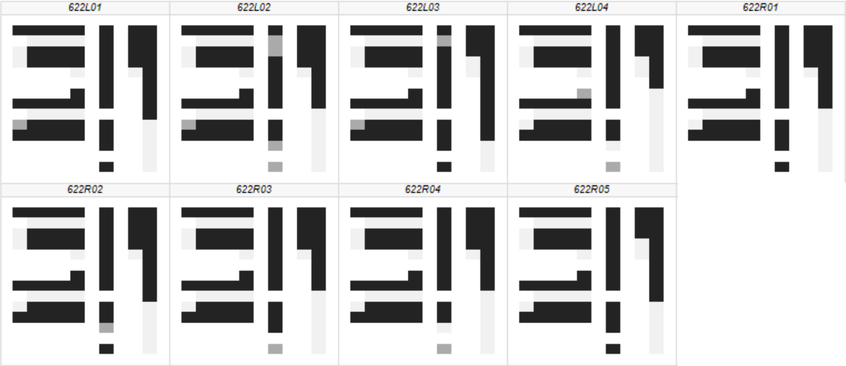

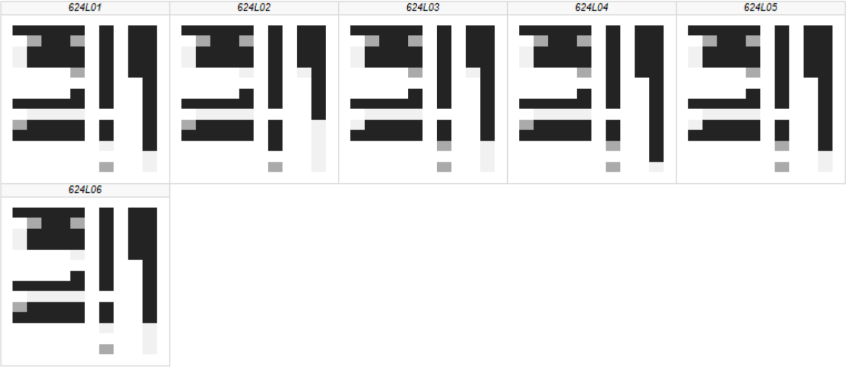

Supplement: Supplementary file 4 [file mmc4.pdf]
